# A Formable Wood‐Based Phase Change Materials with Enhanced Mechanical Properties and Thermal Efficiency for Smart Building

**DOI:** 10.1002/advs.202519262

**Published:** 2025-11-25

**Authors:** Ya Zhou, Yingfeng Zuo, Yuhang Ma, Ping Li, Dawei Zhao, Yiqiang Wu

**Affiliations:** ^1^ School of Materials and Energy Central South University of Forestry and Technology Changsha Hunan 410004 P. R. China; ^2^ Key Laboratory on Resources Chemicals and Materials of Ministry of Education Shenyang University of Chemical Technology Shenyang Liaoning 110142 P. R. China

**Keywords:** cellulose framework, multi‐scale network, phase change materials, smart buildings, thermal management

## Abstract

Solid–liquid phase change materials (PCMs) are capable of absorbing and releasing heat through a reversible phase change process, playing a significant role in thermal protection and energy conservation in smart buildings. However, challenges such as liquid leakage and subsequent declines in mechanical properties persist. Here, a wood‐form‐stable phase change composite (DWTP) is reported, created by regulating the self‐assembly of multi‐active site polyethylene glycol and in situ mineralization. The DWTP features a multi‐scale network formed by gradient hydrogen bonding between cellulose molecules and Si─O─Si/PEG, demonstrating a high enthalpy of 94.73 J g^−1^ and outstanding mechanical tensile strength of 134.42 MPa—the highest reported for any PCM to date. Additionally, this DWTP can support loads exceeding 110 times its weight without deformation and leakage when heated above its phase transition temperature. Following 50 thermal‐cold cycles, the DWTP retains 97.3% of its phase change performance. Outdoor thermal management tests verify that the DWTP cabin achieves a maximum sub‐ambient temperature reduction of 14.1 °C in conditions with an ambient temperature of 50 °C. This biomass DWTP represents a significant advancement in the design of next‐generation sustainable thermal management materials.

## Graphical abstract

A wood‐form‐stable phase change composite featuring a tensile strength of 134.42 MPa, zero leakage under load, and a phase change enthalpy of 94.73 J g^−1^ is developed through a structural biomimicry and dual‐network reinforcement strategy. With its exceptional shape stability and remarkable phase change performance, this phase change composite is well‐positioned to provide innovative solutions for building thermal management.

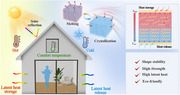

## 1 Introduction

To maintain comfortable indoor temperatures, heating and cooling systems account for ≈50% of a building's total energy consumption. This substantial energy demand not only results in excessive resource use but also significantly contributes to greenhouse gas emissions, thereby impacting the environment.^[^ ^1^, ^2^ ^]^ In response to this issue, researchers have developed various insulating materials, such as aerogels and biofoams, designed to block or slow down heat transfer in targeted areas.^[^ ^3^, ^4^, ^5^ ^]^ Among these, nanoporous structures are especially effective as they restrict the movement of air molecules, a crucial factor in enhancing thermal insulation performance.^[^ ^6^ ^]^ However, the high cost and reliance on energy‐intensive fabrication methods, such as solvent exchange and supercritical drying, have hindered the large‐scale deployment of these materials in practical applications.

Phase change materials (PCMs), at the core of latent heat storage technology, facilitate the absorption and release of thermal energy through reversible phase changes, simultaneously providing energy storage and temperature regulation. This makes them ideal candidates for zero‐energy thermal management solutions.^[^ ^7^, ^8^, ^9^ ^]^ Currently, organic solid–liquid PCMs, including polyethylene glycol (PEG), paraffin, and fatty acids, are of particular interest due to their high enthalpy, energy storage density, low cost, and non‐toxicity.^[^ ^10^ ^]^ Consequently, researchers have developed various organic functional PCMs that show promise for applications in building thermal management,^[^ ^11^, ^12^ ^]^ personal temperature regulation,^[^ ^13^, ^14^ ^]^ solar‐thermal energy conversion,^[^ ^15^ ^]^ and military uses.^[^ ^16^ ^]^ However, challenges such as liquid leakage, significant volume changes, and low mechanical strength continue to impede the practical application of PCMs.^[^ ^17^, ^18^, ^19^, ^20^ ^]^

Wood, one of Earth's most abundant natural biomaterials, has been utilized for millennia, with its unique cellular structure serving as a blueprint for advanced material design.^[^ ^21^, ^22^, ^23^, ^24^ ^]^ Compared to synthetic porous materials, wood offers superior sustainability, biodegradability, and ease of surface modification. Its naturally evolved hierarchical pore connectivity creates a Murray network structure that optimizes mass transfer pathways.^[^ ^25^, ^26^, ^27^, ^28^ ^]^ This makes wood an ideal carrier for energy storage materials, especially through high‐temperature heat treatment or lignin removal processes.^[^ ^29^, ^30^ ^]^ However, these pretreatments, such as delignification or carbonization, can significantly degrade the mechanical properties of the wood.^[^ ^31^ ^]^ Moreover, the mechanical integrity of phase change composites relies on the synergistic contributions from both the supporting material and the encapsulated PCMs. When ambient temperatures exceed the phase transition temperature, the PCMs transition from a rigid to a soft state, resulting in a mechanical mismatch with the rigid support material. This mismatch can lead to phase separation and a continuous decline in mechanical performance, posing potential safety hazards when these materials are utilized in load‐bearing applications.^[^ ^32^, ^33^ ^]^

**Figure 1:**
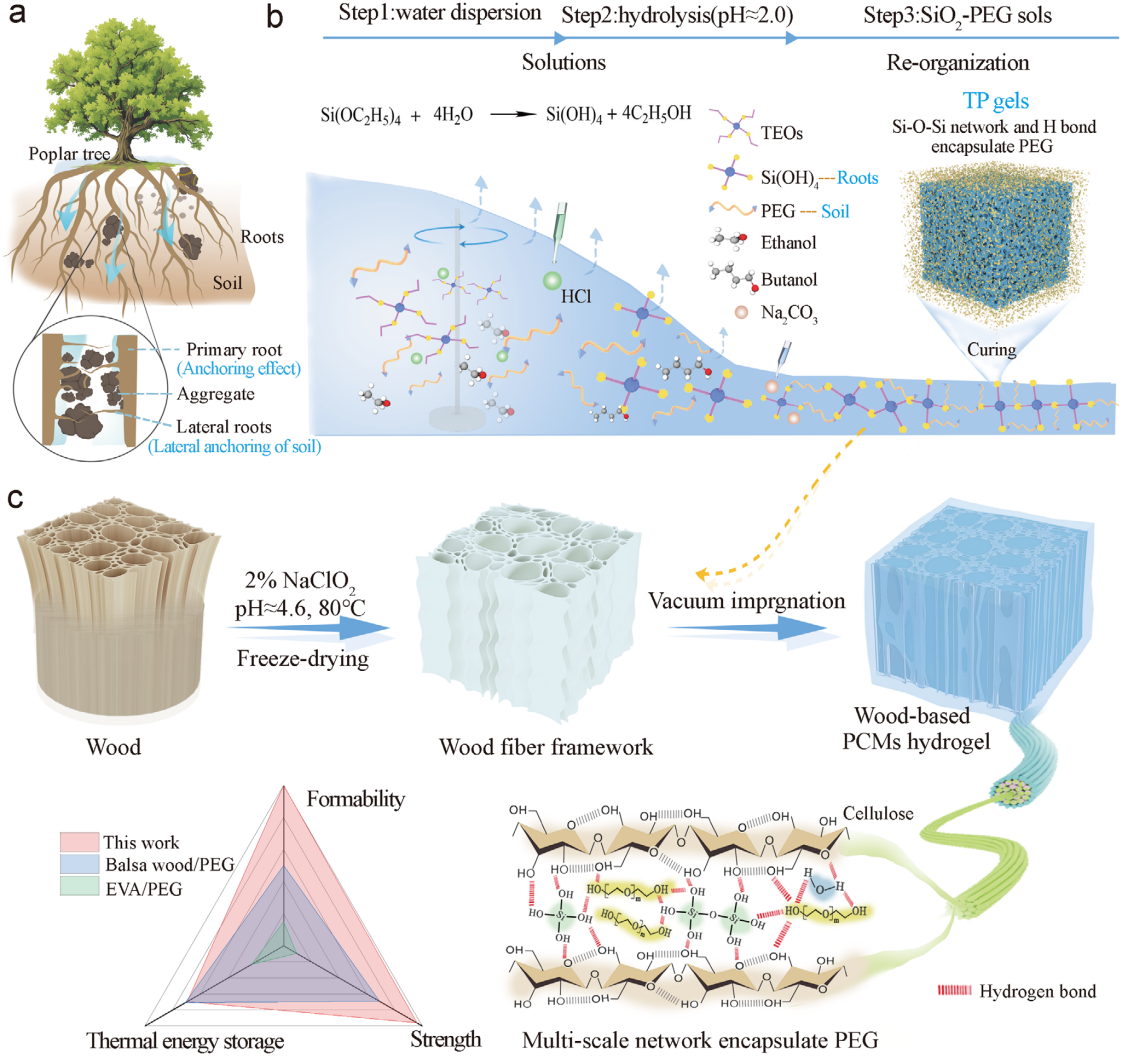
Preparation process and mechanism of DWTP. a) Mechanism of root‐soil interface interlocking. b) Synthesis route and mechanism of TPPCS. c) Structural design of wood‐based dual network phase change composites inspired by root‐soil interface interlocking.

Here, we introduce a highly active siloxane short‐chain structure into PEG through simple hydrolysis, successfully constructing a secondary network within the wood using self‐assembly and in situ mineralization. This process results in the development of a high‐strength, stable biomass phase change material (noted as DWTP). Inspired by the mechanism of nutrient sequestration in plant roots (**Figure** **1**a), we utilized tetraethyl orthosilicate (TEOs), a hydrolysis product yielding silicon hydroxide (Si(OH)_4_), and treated it as lateral roots. This was mixed with PEG, which served as the soil, to create a homogeneous and stable phase change sol (TPPCS, Figure 1b). Subsequently, using delignified wood as a template—acting as the main root—we efficiently loaded the phase change sol into the wood fiber backbone via vacuum impregnation (Figure 1c).

In this system, Si(OH)_4_ functions dually: it provides structural support for the PEG matrix and forms a 3D supramolecular network with the active hydroxyl (─OH) sites of PEG and wood cellulose fibers through hydrogen bonding. This multi‐scale network endows the DWTP with exceptional leakage resistance and mechanical strength. When subjected to temperatures above the phase change threshold of 80 °C, the DWTP can support over 110 times its own weight without deformation or leakage, effectively addressing the common issues of deformation and leakage found in traditional PCMs at elevated temperatures. These improved properties render the DWTP highly promising for applications in energy‐efficient buildings and thermal management, with its breakthrough mechanical performance significantly enhancing its value in engineering applications.

## 2 Results and Discussion

### 2.1 Synthesis and Controlled Exothermic Properties of TPPCS

**Figure 2:**
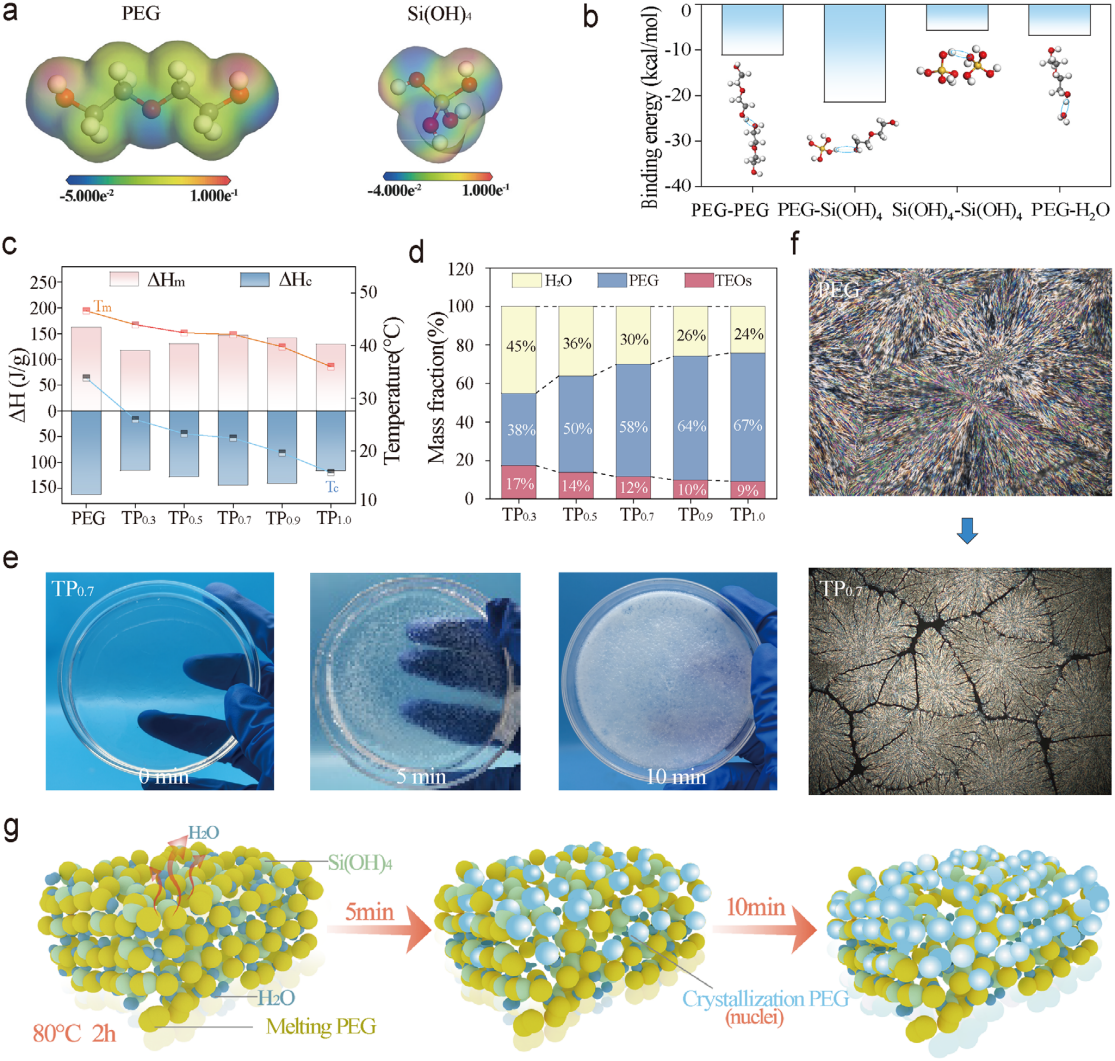
Synthesis and properties of TP. a) ESP distribution of Si(OH)_4_ and PEG units. b) Binding energy between different molecules in TPPCS. c) The crystallization enthalpy (ΔH_c_), the melting enthalpy (ΔH_m_) and phase change temperature of TP. d) Composition of TP. e) Experimental photographs of thermally induced crystallization of TP. f) POM images of PEG, TP_0.7_ at 25 °C. g) Thermally induced crystallization mechanism of TP.

In this study, a shape‐stabilized structure for PEG was developed by mimicking the interlocking mechanism between tree roots and soil. TEOs were blended with PEG and subsequently hydrolyzed to generate the active species Si(OH)_4_ (polysilicic acid). The surface Si─OH groups acted as adsorption sites, forming hydrogen bonds with the terminal ─OH groups of PEG, thereby effectively anchoring the PEG within the matrix (Figure S1, Supporting Information). This gradient hydrogen‐bonding interaction delayed the condensation and phase separation of the PEG/Si(OH)_4_ sol, resulting in the formation of a homogeneous and stable phase‐change colloid.^[^ ^34^ ^]^ To verify the formation mechanism of the TPPCS, the intermolecular binding energies between Si(OH)_4_ and PEG were analyzed through theoretical calculations, as shown in **Figure** **2**a. The electrostatic potential (ESP) distribution plots reveal strong electrostatic interactions between Si(OH)_4_ and PEG, with the attraction potentials predominantly localized around the ─OH groups of both components. The calculated binding energy reaches −21.4 kcal mol^−1^, which is significantly higher than those of other intermolecular hydrogen bonds present in TPPCS (Figure 2b; Figure S2, Supporting Information). These findings elucidate the molecular interaction mechanism between the structural units of TP sols and support the proposed formation theory.

TPPCS were initiated at elevated temperatures to achieve PEG stereospecificity through Si(OH)_4_ condensation to form a Si─O─Si network and hydrogen bonding synergies (Figure S3, Supporting Information). In general, polymerization restricts the mobility of PEG molecular chains. As the proportion of TEOs increases, the density of the Si─O─Si network correspondingly rises (Figure S4, Supporting Information). This results in a greater number of Si─O─Si structures being embedded within the PEG matrix, along with an increased number of hydrogen bonds. Consequently, the phase transition enthalpy of the TP composites gradually decreases due to the reduced freedom of PEG chain motion (Figure 2b,c; Figure S5, Supporting Information).^[^ ^35^ ^]^ The melting enthalpy reached a maximum value of 146.67 J g^−1^ when the molar ratio of TEOs to PEG was 1:0.7. This result also supports the binding energy calculations, confirming that the hydrogen bonding between Si(OH)_4_ and PEG is stronger than that between PEG chains. This is attributed to the competitive substitution of PEG crystalline domains by Si(OH)_4_, which disrupts PEG‐PEG interactions and enhances Si(OH)_4_‐PEG interactions. In addition, this hydrogen‐bonding competition effect suppresses the crystallization of PEG, resulting in TP gels that remain transparent after being stored at room temperature (10–35 °C) for 60 days (Figure S3, Supporting Information). This observation suggests that the TP gels can maintain a long‐term supercooled state, enabling stable thermal energy storage. However, after entering winter (room temperature 0–10 °C), we found that samples with TP_X>0.7_ turned white and had spontaneously crystallized. In contrast, samples with TP_X ≤0.7_ remained transparent and continued to supercool for up to 120 days or more.

Considering the chemical composition, this can be attributed to the intermolecular interactions (e.g. hydrogen bonding) between PEG, Si(OH)_4_ and water, which hinder hydrogen bonding between PEG molecules and form crystallization energy barrier, limiting the formation of an ordered crystal structure of PEG.^[^ ^36^, ^37^ ^]^ The increase in supercooling of samples with TP_X ≤0.7_ may be due to the increase in the ratio of water and TEOs in the reaction system, the formation of enhanced hydrogen bonding network, and the increase in the crystallization energy barrier of PEG. To verify this conjecture, we placed the supercooled samples in an oven at 80 °C for 1 h, then observed the crystallization process and recorded time. As shown in Figure 2e, TP_0.7_ gels showed many white dotted crystals inside after 5 min, and the area gradually expanded. And after 10min, the sample has been basically crystallized and changed from a transparent gel to a white composite material. To further investigate the crystallization behavior of the TP composites, the crystalline morphologies of PEG and TP_0.7_ were examined using POM, which revealed clear evidence of crystal growth (Figure 2f; Figure S6, Supporting Information). The POM images of PEG and TP_0.7_ at 25 °C show a cross‐extinction spherical crystal morphology, indicating that TP_0.7_ has a similar crystal structure to PEG. However, the spherical crystal size and crystallization rate of TP_0.7_ are smaller than those of PEG, and there are “cracks” in the spherical crystal. The “cracks” may be caused by the amorphous SiO_2_ network and hydrogen bonding network formed through Si(OH)_4_ condensation. This structural disruption further supports the successful construction of the root‐soil‐inspired interlocking architecture (Figure S7, Supporting Information).

In addition, supercooling primarily arises from the strong hydrogen‐bond donor and acceptor characteristics of water molecules, which form hydrogen bonds with the ─OH groups of PEG molecular chains. This interaction creates a barrier between PEG chains, thereby inhibiting the aggregation of crystalline domains and suppressing PEG crystallization. Heating triggers water evaporation, releasing PEG molecular chains from aqueous hydrogen‐bonding interactions. The PEG chains reorganize via intermolecular hydrogen bonding to establish crystal nuclei, driving accelerated PEG crystal growth and crystallization (Figure 2g). This demonstrates that the TP composite phase change material can achieve prolonged supercooling by adjusting reactant ratios, where water molecules act as molecular “locks” to delay phase transition, trapping phase change latent heat within the material. Controlled heat release is subsequently enabled through a thermal evaporation‐induced “key” mechanism, realizing programmable latent energy liberation in the composite phase change system. Considering the anti‐leakage property, enthalpy of phase change, and long‐term stability of TP composites, we chose TP_0.7_ (hereafter referred to as TP, and the term DWTP in the manuscript refers specifically to DWTP_0.7_, unless otherwise stated) as a functional substance to be combined with DW to form a secondary network in wood to construct a composite PCM encapsulated by a dual network.

### 2.2 Morphology and Structure of DWTP Phase Change Composites

**Figure 3:**
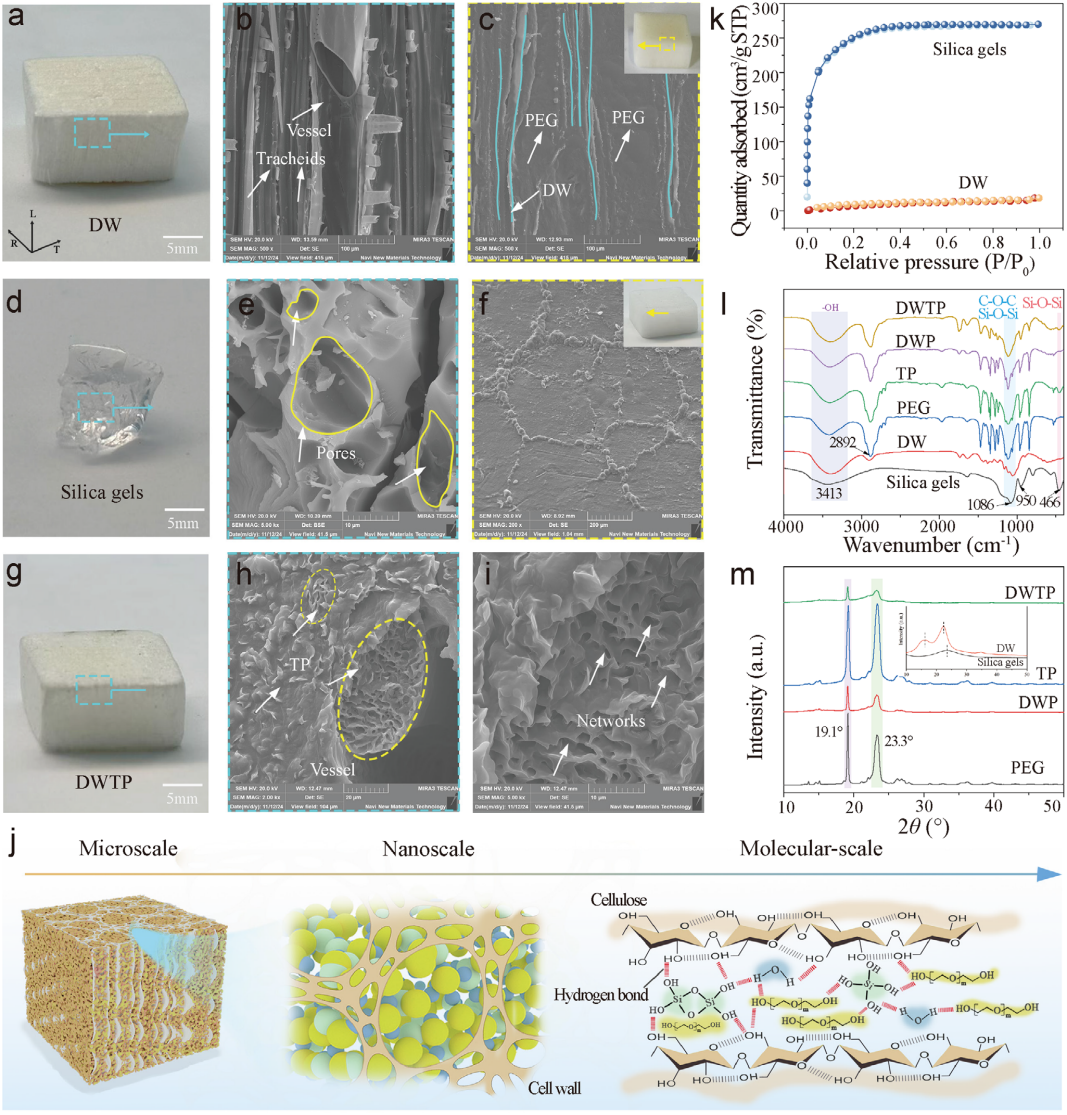
Morphology and structure of phase change composites. a,d,g) Photograph of DW, silica gels, DWP. SEM images of b) DW, c) DWP, e) Silica gel, f) TP, h,i) DWTP. j) Multiscale structure of the DWTP dual network composite phase change material. k) DW and silica gel N_2_ adsorption and desorption isotherms. l) Infrared spectra of silica gel, DW, PEG, TP, DWP, and DWTP, and m) XRD patterns of silica gel, DW, PEG, TP, DWP, and DWTP.

The superiority of DWTP was verified by testing single/dual network phase change composites against each other. The microscopic morphology of the samples was tested using SEM. As shown in **Figure** **3**a,b, PW after delignification retains the porous structure of natural wood and more reactive ─OH groups are exposed,^[^ ^27^, ^38^ ^]^ which can provide reaction and anchoring sites for PEG. Comparison of the sample morphology reveals that the framework materials and phase change substances of DWP (Figure 3c) and TP (Figure 3f) are uniformly embedded. Unlike DWP and TP, a double network structure was clearly observed in DWTP (Figure 3g–i), indicating that the secondary network was successfully formed in the vessels and tracheids (primary network) of DW and was tightly articulated with the DW cell wall, suggesting a good interfacial compatibility between the PCMs and the framework material. To further illustrate the interaction between the framework material and PCMs, we investigated the structure of DW and Silica gels using BET tests. As shown in Figure 3k and Figure S8 (Supporting Information), them show the N_2_ adsorption–desorption isotherms and pore size distributions of DW and Silica gels, respectively. Compared to DW, the in situ synthesized silica gels exhibit a significantly larger specific surface area (up to 879.6 m^2^ g^−1^). This property indicates that the silica gel not only possesses a larger effective contact area, but at the same time its surface exposes many active sites favorable for the reaction with PEG and DW. Such advantage enables it to simultaneously act as an encapsulation carrier for PEG and a bridge to connect DW, thus forming an interconnected porous network structure within DWTP.

To further confirm the formation and influence of the dual network structure, it was analyzed using FTIR and XRD. The FTIR spectrum of Silica gels, DW, PEG, TP, DWP, and DWTP are shown in Figure 3l. On the FTIR spectra of Silica gels, 3413, 1086, 950, 802, and 466 cm^−1^ are attributed to the stretching vibration of ─OH, asymmetric stretching vibration of Si─O─Si, symmetric stretching vibration of Si─OH, symmetric stretching vibration of Si─O─Si, and bending vibration of Si─O─Si, respectively.^[^ ^39^ ^]^ The characteristic absorption peaks of PEG appeared at 3424, 2892, and 1122 cm^−1^, which were attributed to the vibrations of ─OH, ─CH, and C─O─C bonds, respectively.^[^ ^40^ ^]^ Compared with pure PEG, the typical characteristic peaks of silica gels appeared in TP and DWTP, indicating the successful formation of Si─O─Si cross‐linked network within the phase change composites. In addition, the characteristic ─OH peaks of TP, DWP and DWTP were broadened and shifted to lower frequencies, indicating the formation of multiple hydrogen bonding networks between silica gels, DW and PEG (Figure 3j). However, in addition to the characteristic peaks of the framework material and PEG, no new chemical structures have formed in the DWTP, indicating that only physical interactions occur between PEG and the framework material.^[^ ^41^ ^]^

The crystallization properties of phase change composites directly determine the phase change properties such as phase change temperature and enthalpy. According to the XRD plots of the samples (Figure 3m), PEG shows obvious diffraction peaks at 2θ of 19.1° and 23.3°, which correspond to (120) and (112) crystalline surfaces, respectively. Meanwhile, the lowest intensity of the diffraction peaks can be clearly seen for DWTP, which indicates a decrease in the crystallization properties of PEG. The wood encapsulates the TPPCS on a macroscopic scale by capillary action, and the Si(OH)_4_ monomer or multimer in the sols condenses in the wood to form polysiloxane chains, which spatially extend the region of confinement to the PEG. In addition, Si(OH)_4_, PEG forms hydrogen bonds with reactive ─OH on the wood cellulose framework, further restricting the movement of the PEG molecular chain on a molecular scale. These two reasons led to a decrease in the degree of crystallization of DWTP. In addition, similar crystal diffraction positions to those of pristine PEG were observed in the XRD curves of TP, DWP, and DWTP, which suggests that the crystalline phases of the above composites originate from the PEG molecular chains, and that the physical constraints do not affect the crystal structure of the PEG molecular chains.^[^ ^42^, ^43^ ^]^ It was further confirmed that all components in TP, DWP and DWTP are bound together by physical interaction.

### 2.3 Leakage Protection and Mechanical Properties of DWTP Phase Change Composites

**Figure 4:**
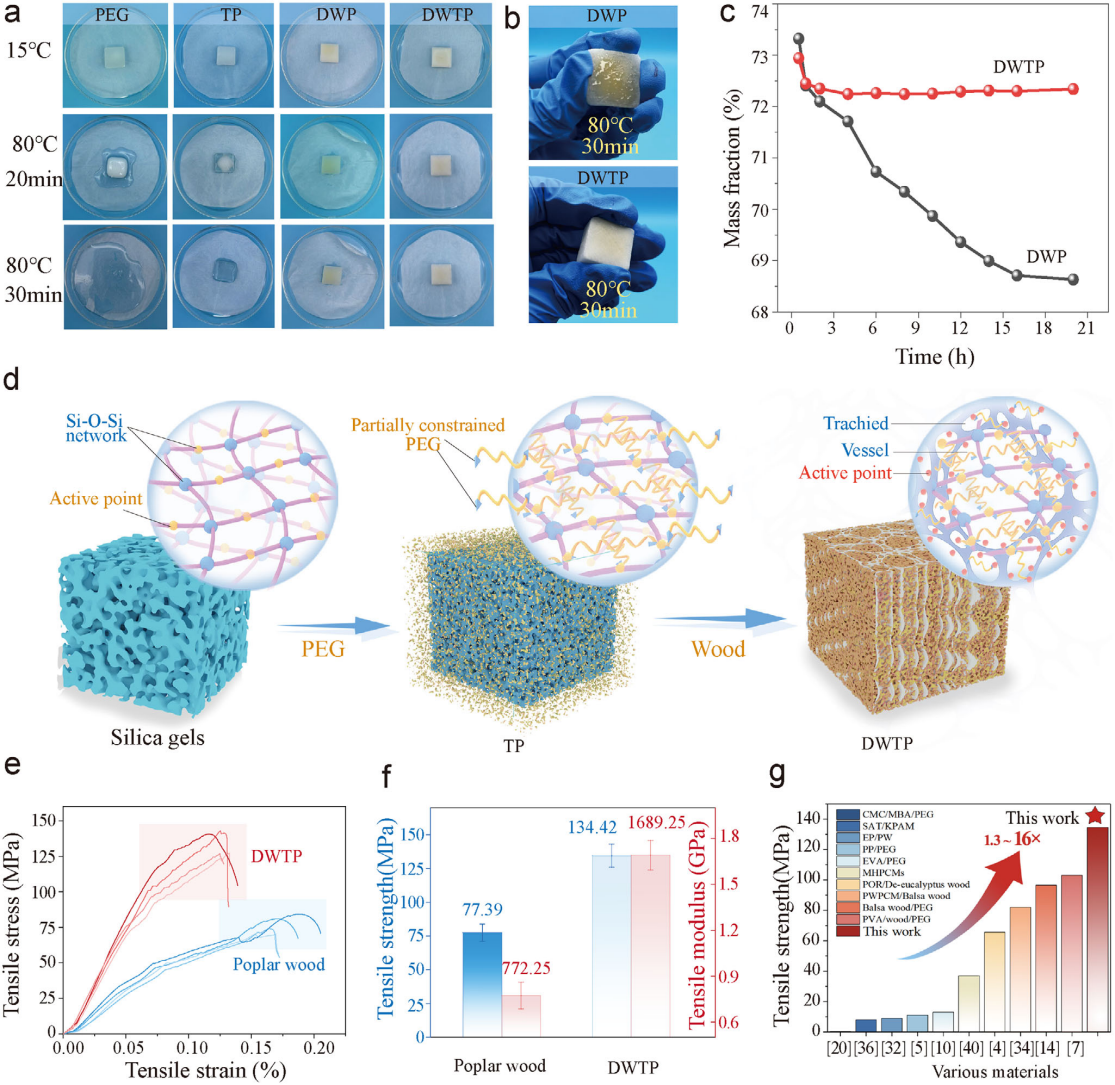
Leakage prevention properties and mechanism of phase change composites. a) Photographs of PEG, TP, DWP, and DWTP heated at 80 °C. b) Extruded leakage diagrams of DWP and DWTP after heating. c) Leakage curves of DWP and DWTP. d) Leakage prevention mechanism of DWTP. e) Stress–strain curves of PW and DWTP. f) Tensile strength and elastic modulus of PW and DWTP. g) Tensile strength and modulus of elasticity compared with the existing PCMs.

Leakage resistance and mechanical properties are the prerequisites for the large‐scale application of solid–liquid PCMs. To verify the leakage resistance of the dual network composites, the leakage resistance of the composite PCMs was evaluated in multiple dimensions as shown in **Figure** **4**a–c. As shown in Figure 4a, PEG gradually melts and transforms from solid to liquid state when it is heated at 80 °C for 20 min. In contrast, the TP, DWP, and DWTP exhibit solid‐solid phase transition behavior during heating due to capillary action and Si─O─Si network that has a restraining effect on PEG molecules. However, in TP, when PEG transformed into liquid, TP gradually became transparent and basically maintained shape stability, but a small amount of leakage still existed. This occurs because a portion of the PEG is not completely confined by the 3D network and exists in a semi‐free state. As the temperature increases, the partially confined PEG molecular chains are released from the active sites, transitioning into a disordered state that causes PEG to leak (Figure 4d).

For the DWTP, the active groups (e.g., ─OH, ─COOH) of the wood fiber skeleton anchored the TPPCS in the DW, and some of the restricted or free‐state PEG molecular chains were confined by the DW pores and active sites, which effectively prevented the leakage of PEG in the DWTP. In addition, the sample loaded with 500 g and heated for 30 min was extruded (Figure 4b; Figure S9, Supporting Information), and it was found that the PEG in the DWP leaked significantly, but there was no change in the shape of the DWTP, and there was no PEG leaked from the surface, which confirmed the excellent leakage‐proof performance of the DWTP at the melting temperature (T_m_). To further investigate the anti‐leakage performance of the composite PCMs above the T_m_ for a long time, the mass changes of DWP and DWTP heated at a temperature of 80 °C for 20 h were tested, as shown in Figure 4c. It can be observed that DWTP exhibits more stable mass changes with significantly less leakage, showing almost no leakage. This is mainly attributed to the synergistic effect of the DW cellulose skeleton and the Si─O─Si network, as well as the capillary action of the DW pores.

To verify the mechanical properties of DWTP, the samples were tested in tensile. Figure 4e,f shows the stress–strain curves of the samples and their tensile strength. The results showed that the tensile strength and modulus of elasticity of DWTP were significantly higher than PW, which were 134.42 and 1689.25 MPa, respectively, and ≈1.74 and 2.19 times higher than PW. Compared with PW, DWTP has a dense network structure, including cellulose network, Si─O─Si network, and PEG intermolecular chain network, which effectively improve the tensile properties of the composites through hydrogen bonding and entanglement effects.^[^ ^40^, ^44^ ^]^ Among them, the Si─O─Si network is a rigid structure, which effectively enhances the integrity of the composites. Notably (Figure 4g; Table S2, Supporting Information), DWTP exhibits significantly higher tensile strength and modulus of elasticity, which are ≈1.3–16 times higher than those of other solid–liquid PCMs and wood‐based shaped PCMs. The enhancement of these properties makes DWTP promising for green building applications for efficient thermal management during building operation.

### 2.4 Phase Change Behavior and Thermal Stability of DWTP Phase Change Composites

**Figure 5:**
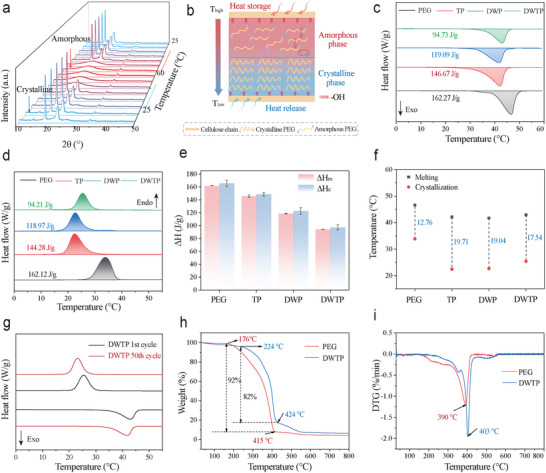
Phase change properties and thermal stability of samples. a) In situ XRD during melting and crystallization of DWTP. b) DWTP maintains temperature equilibrium by absorbing heat in a phase transition. c) Melting and d) Crystallization DSC curves of PEG, TP, DWP, and DWTP. e) The melting enthalpy and crystallization enthalpy of PEG, TP, DWP, and DWTP. f) Supercooling of PEG, TP, DWP, and DWTP. g) DSC curves of DWTP before and after 50 cycles. h) TG and i) DTA curves of PEG and DWTP.

To evaluate the phase transition behavior of DWTP during melting and crystallization, the samples were subjected to in situ XRD. With the temperature increasing, the PEG component of DWTP undergoes a phase transition, as evidenced by the disappearance of its crystal diffraction peaks and the appearance of broader diffraction signals (**Figure** **5**a). It indicates that the PEG molecular chains underwent a transition from a regular arrangement to an amorphous state under external thermal stimulation (Figure 5b). DSC tests revealed the phase transition behavior of PEG, TP, DWP, and DWTP, as shown in Figure 5c–f and Figure S10 (Supporting Information). The results showed that the phase transition temperature can be adjusted by changing the molar ratio of the TPPCS. The T_m_ of the DWTP_x_ (where x represents the molar ratio of PEG, e.g., DWTP_0.3_ to DWTP_1.0_) range from 33.2 to 42.9 °C, and the crystallization temperatures (T_c_) range from 12.7 to 25.4 °C.

In Figure 5e, the melting enthalpy of TP, DWP, and DWTP are all lower than that of PEG (162.27 J g^−1^), with values of 146.67 , 119.09, and 94.73 J g^−1^, respectively. This reduction is attributed to two factors: first, the spatial confinement imposed by the framework material, which restricts the mobility and ordered arrangement of the PEG molecular chains. Second, the dilution effect of the supporting matrix (DW and silica gels), which are not PCM themselves, thereby lowering the overall mass fraction of active PEG in the composite. Besides, the T_m_ and T_c_ of TP, DWP, and DWTP composite PCMs are lower than PEG, and the degree of supercooling is increased (Figure 5f). This is mainly due to the difficulty of regular arrangement of PEG molecular chains under the restriction of the dual network, the retardation of crystal growth, and the decrease in the size of the crystals, resulting in a decrease in the phase transition temperature and enthalpy (Table S3, Supporting Information).^[^ ^45^ ^]^

The thermal cycling performance and thermal decomposition of DWTP are important bases for evaluating its thermal stability. As shown in Figure 5g, compared with the pristine state, the enthalpy of DWTP is basically unchanged after 50 times of thermal cycling, indicating that DWTP has good thermal cycling stability. Figure 5h,i illustrates the thermal decomposition process of DWTP. It can be observed that PEG begins to decompose at 176 °C, reaching its maximum weight loss rate at 415 °C. For the DWTP, thermal decomposition occurs within the temperature range of 224 to 551 °C. However, in the interval of 424 –551 °C, the decomposition rate slows down. This indicates that the Si─O─Si network within DWTP partially protects PEG from decomposition, thereby increasing the decomposition temperature of the phase change composite by 48 °C. The slower thermal decomposition rate may be attributed to the carbonization of cellulose and the decomposition of a small amount of Si(OH)_4_ in DWTP.^[^ ^46^ ^]^ In conclusion, DWTP phase change composites have better heat resistance than PEG.

### 2.5 Temperature Regulation Capability of DWTP Phase Change Composites

**Figure 6:**
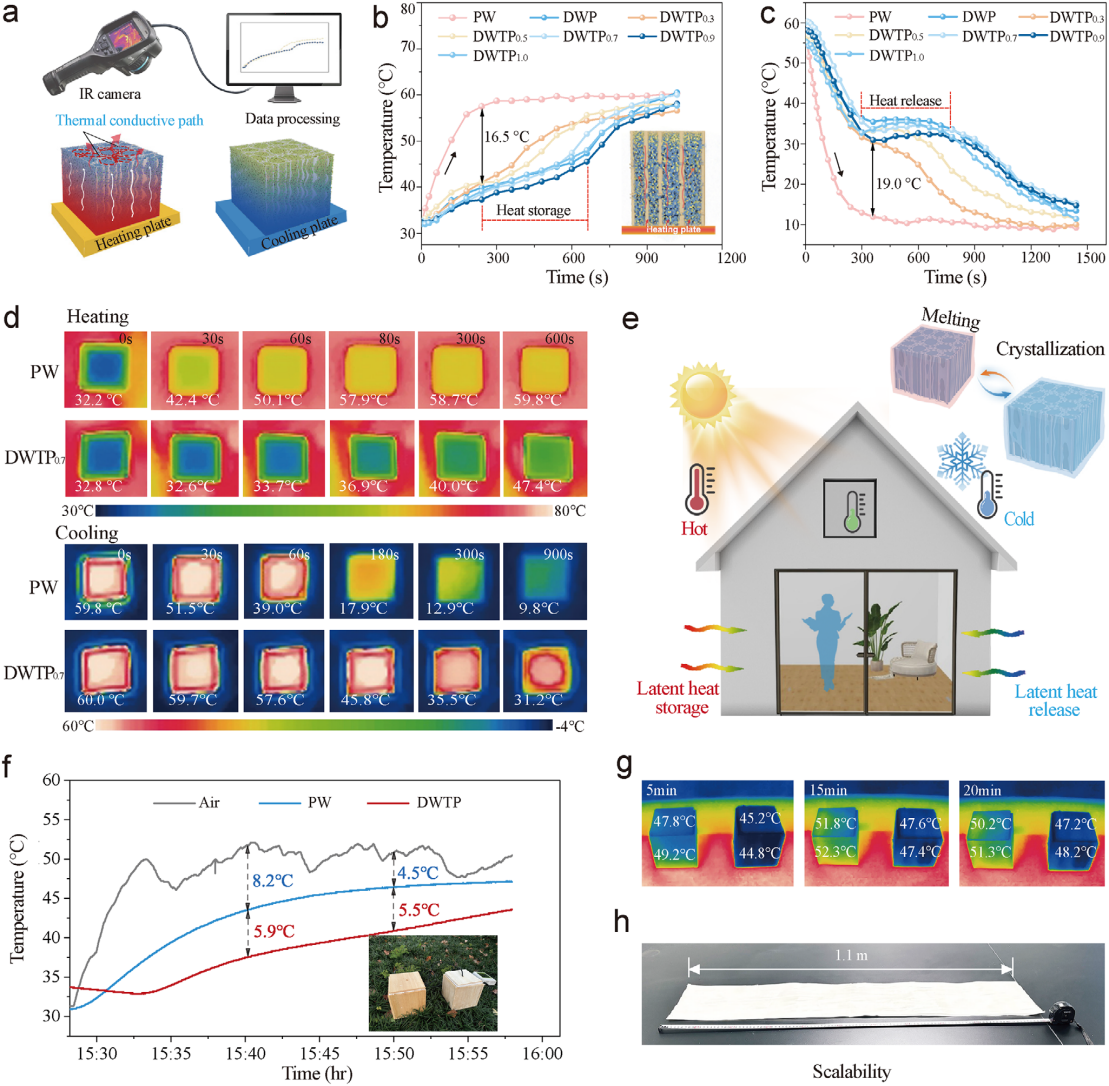
Temperature regulation capability of samples. a) DWTP heating and cooling test model. b) Temperature profiles of DWP and DWTP samples during the heating and c) the cooling process. d) Thermal infrared imaging of DWP and DWTP samples during the heating and cooling process. e) The application potential of DWTP in building temperature regulation. f) Temperature response curves within 30 min under solar irradiation. g) Infrared thermographs of DWTP and PW surfaces. h) Large‐scale DWTP prepared from natural wood.

To study the potential of DWTP in building thermoregulation. The temperature regulation performance of the samples in heating and cooling were tested by placing the samples on a platform at a temperature of 80 and −4 °C, and taking digital photos with an IR camera (**Figure** **6**a). The results showed (Figure 6b) that the temperature of PW increased rapidly with heating time increasing, while DWP and different groups of DWTP at 42–55 °C, the temperature of the sudden change and the formation of an obvious temperature buffer, and in the heating process of the sample's surface temperature is always lower than 80 °C, up to 62 °C after the equilibrium no longer rise. This is mainly attributed to the transformation of the PEG inside the composite into a molten phase that stores more heat.^[^ ^47^ ^]^ During the cooling process (Figure 6c), the temperature of PW was reduced from 60 to ≈10 °C in 5 min. In contrast, it took ≈25 min for the DWTP temperature to be reduced to 10 °C. In addition, the DWTP showed a clear temperature plateau in the range of 30–40 °C, which was maintained for ≈10 min, indicating that the DWTP has a great potential for temperature regulation. In addition, the thermal properties of DWTP can be visualized through the sample surface color comparison (Figure 6d; Figures S12 and S13, Supporting Information).

Notably, as the proportion of TEOs increases, the thermal storage time of DWTP decreases. This may be due to the decrease in the proportion of PEG in the composites, which restricts the movement of molecular chains in the network, leading to a decrease in the enthalpy of phase change. But the heating rate of DWTP samples is faster than that of DWP, and it takes less time to reach the phase transition temperature point. This is mainly because the TPPCS is filled in the wood to achieve wood densification and form a continuous heat conduction path (Figure S14, Supporting Information). Meanwhile, the Si─O─Si network embedded in PEG also facilitates the construction of heat conduction pathways and enhances the thermal response rate of phase change composites (Figure 6e). To verify the thermal regulation performance of DWTP under atmospheric conditions, a 100 × 100 × 100 mm^3^ chamber was constructed for thermal management tests, with the PW group serving as the control. As illustrated in Figure 6f, the DWTP chamber exhibited a maximum sub‐ambient temperature drop of 14.1 °C under atmospheric conditions.

In contrast, the PW chamber showed a significantly smaller maximum reduction of only 8.2 °C below the ambient temperature. Furthermore, the outer surface temperatures of the chambers were monitored using infrared thermography, as presented in Figure 6g. At the same time interval, the surface temperature of DWTP was 2.5–4.9 °C lower than that of PW. This cooling effect is primarily attributed to the synergistic contribution of radiative cooling from delignified wood (Figure S15, Supporting Information) and the heat absorption capacity of PEG. In addition, large‐scale fabrication of DWTP was successfully achieved (Figure 6h). These performance enhancements provide practical implications for the application of DWTP in building thermal management and temperature control.

## 3 Conclusion

In summary, we propose a structural biomimetic and dual‐network reinforcement strategy to develop a high‐strength, temperature‐regulating wood‐form‐stable phase change composite. By blending TEOs with PEG, a phase change precursor with multiple active sites was obtained through hydrogen‐bond competition effect. Integrating this precursor with the cellulose framework through self‐assembly and in situ mineralization, a multiscale interlocking structure composed of cellulose molecules, Si─O─Si networks, and hydrogen bond networks was constructed, thereby anchoring the PEG. Consequently, DWTP exhibited robust mechanical properties (134.42 MPa) and leakage resistance (no leakage under load at 80 °C melt temperature). Furthermore, DWTP maintained 97.3% of its thermal storage capacity after 50 thermal‐cold cycles. Testing demonstrated remarkable temperature regulation in DWTP due to PEG's thermal/cold buffering effect. These properties offer practical opportunities for applications in energy‐efficient buildings and thermal management. These characteristics provide practical opportunities for its application in energy‐efficient buildings and thermal management systems.

## 4 Experimental Section

### Materials and Chemicals

Poplar (*Populus tomentosa Carr*), 0.43–0.57 g cm^−3^ density, was acquired from Chenzhou (Hunan Province, China). Sodium chlorite (NaClO_2,_ ≥80%) were supplied by Shanghai Macklin Biochemical Co., Ltd., China. Acetic acid (CH_3_COOH, AR), hydrochloric acid(HCl, AR), sodium carbonate (Na_2_CO_3_, AR), tetraethyl orthosilicate (TEOs, AR), and polyethylene glycol(PEG, 1500 g mol^−1^)were purchased from Sinopharm Chemical Reagent Co., Ltd, China. Ethanol (CH_3_CH_2_OH, AR) was bought Tianjin Hengxing Chemical Reagent Manufacturing Co., Ltd, China. Ultrapure water was used in all experiments.

### Preparation of DW

A 2 wt.% NaClO_2_ solution was first prepared, and the pH was adjusted to 4.6 using acetic acid titration. Subsequently, a piece of poplar wood (PW) with dimensions of 20 mm (R)× 20 mm (T) × 10 mm (L) was immersed in the solution and treated at 78 °C in a water bath for 16 h. The resulting delignification wood was then soaked in 50 wt.% ethanol solution, with the solution being replaced multiple times until it became colorless, ensuring the complete removal of residual chemicals. Finally, the samples were pre‐frozen at −50 °C for 12 h and then freeze‐dried under conditions of 0–10 Pa, obtaining wood aerogel.

### Preparation of DW/TEOS/PEG (DWTP)

TEOs and PEG were mixed according to different molar ratios (shown in Table S1, Supporting Information), and the mixture was stirred in water bath at 50 °C for 1h until the solution became transparent. Then, the pH was adjusted to 4.03–5.14 by adding an appropriate amount of Na_2_CO_3_ solution, and the mixture was removed after stirring for 4 h and aged overnight at room temperature to obtain the TEOs/PEG phase change sol (TPPCS). The DW was immersed in the TPPCS by vacuum impregnation (−0.095–−0.1 MPa) for 3 h, preparing the dual‐network composite PCM. To demonstrate the superiority of the dual‐network structure, two single‐network samples were prepared as controls, with leakage rate as the primary factor for process selection, and were labeled as DWP, TP_x_, and DWTP_x_ (x represents the molar amount of PEG).

## Conflict of Interest

The authors declare no conflict of interest.

## Author Contributions

Y.Z., P.L., and D.Z. supervised the project and designed the experiments. Y.Z. and Y.M. carried out most of the experiments. Y.M. participated in DFT simulation analysis and microscopic morphology analysis. Y.Z. wrote the paper. Y.Z., D.Z., and Y.W. collectively reviewed the paper. All authors discussed the results and commented on the manuscript.

## Supporting information



Supporting Information

## Data Availability

The data that support the findings of this study are available from the corresponding author upon reasonable request.
