# Supplementary material for: A Formable Wood‐Based Phase Change Materials with Enhanced Mechanical Properties and Thermal Efficiency for Smart Building
Source: Adv Sci (Weinh). 2025 Nov 25;13(8):e19262. doi: 10.1002/advs.202519262 (PMC12884778; doi:10.1002/advs.202519262)
Supplement: Supplementary file 1 — Supporting Information [file ADVS-13-e19262-s001.docx]

**SUPPORTING INFORMATION**

**I. Supplementary Method**

**Characterization:** The pore structure of the samples was analyzed using a specific surface area and pore size analyzer (BET, ASAP 2020, Micromeritics, USA) to test the adsorption and desorption properties of the samples under N_2_. The chemical structures of the samples were characterized using a Fourier transform infrared spectrometer (FTIR, Nicolet iS50, Thermo Fisher, US) in the wavelength range of 4000~ 400 cm^-1^. The crystal structure of the samples was determined in the range of 10°~80°using CuKa rays (λ= 1.541 Å) at 40 kV and 100 mA by x-ray diffractometer (XRD, D8A A25, Bruker, America). The crystalline morphology of the samples was observed by polarized light microscopy (POM, DM2700, Leica, Germany). The morphology and microstructure of the samples were analyzed using scanning electron microscopy （SEM，MIRA3 LMH，TESCAN, Czech Republic）and x-ray energy spectrometry (EDS, One Max 20, Oxford, UK). The phase-change behavior of the samples was measured from −15°C to 60 °C using a Differential Scanning Calorimetry (DSC, TA 250, TA Instruments, USA) at a scanning rate of 5 °C min⁻¹. The phase change enthalpy (ΔH) value was obtained through integration, specifically the area under the endothermic peak (melting) or exothermic peak (crystallization) bounded by the baseline in the DSC curve. Thermal cycling performance data were collected in the DSC instruments at the 1st and 50th cycles. To evaluate the thermal response rate of the samples, a thermal infrared imager （HM-TPK20-3AQF/W, HIKMICRO, China）was utilized to record the temperature changes of the samples. The thermal stability of the samples was tested in the temperature range of 30 ~ 800 °C using a thermogravimetric analyzer (TGA, TGA3+, METTLER TOLEDO, Switzerland) protected by N_2_ at a scan rate of 10 °C/min. The tensile strength of poplar and DWTP was tested according to GB/T 1040 using a universal mechanical testing machine (DR-508A, Dongguan Dongri Instrument Co. Ltd., China).

The leakage-rate test: Each samples was weighed and placed on filter paper, then put in an oven at 80 °C. At regular intervals the specimen was reweighed to record its mass, and the filter paper was replaced. The encapsulation rate (W, %) was calculated using the following equation:

W = (M_1_−M_0_)/M_0_×100 (1)

Where the M_0_ is the weight of wood-based frame; the M_1_ is the weight of samples after the leakage test.

Theory Calculation: In Materials Studio2023, PEG, Si(OH)_4_, and H_2_O molecules was constructed and geometrically optimized using the DFT method of the Dmol3 module. The exchange-correlation functional was described by the generalized gradient approximation (GGA) with BLYP parameterization. The convergence tolerances for energy were set to 1×10⁻⁵ Ha/Å. The optimized geometries of the molecules were utilized to calculate the binding energy (E_B_) by the following equation:

E_B_ = E_total_ – E_1_- E_2_ (2)

where E_total_ represents the total energy of the molecular composite, E₁ and E₂ are the energies of molecule-1 and molecule-2, respectively.” in revised supplementary information.

**II. Supplementary Figures**

**
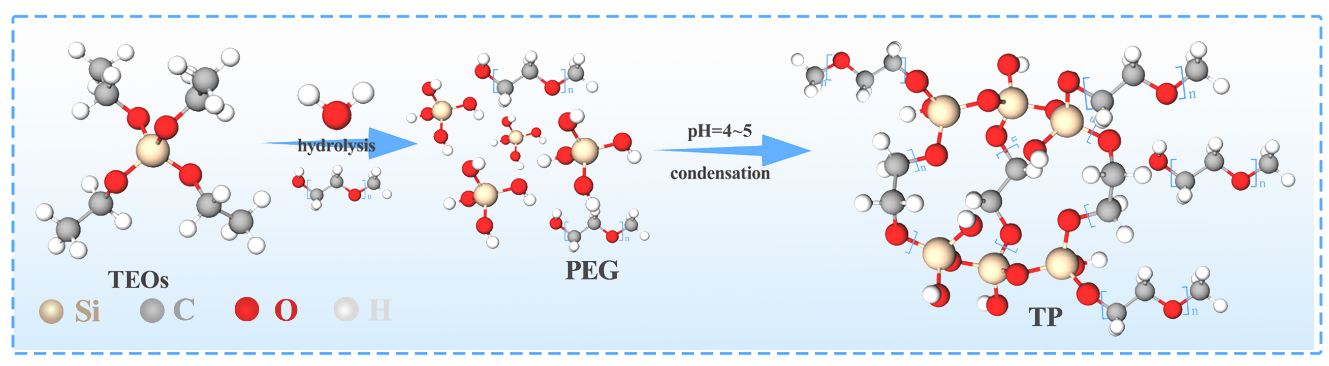
**

**Figure S1.** Mechanism of TP phase change composite formation. TEOs is a commonly used silicon-based precursor that undergoes hydrolysis to form reactive silicic acid (polysilicic acid), which contains abundant surface Si–OH active sites. These sites serve as adsorption points that form hydrogen bonds with the terminal –OH groups of PEG, thereby capturing PEG molecules. This interaction delays the condensation and phase separation of the PEG/Si(OH)₄ sol, resulting in a uniform and stable colloid.


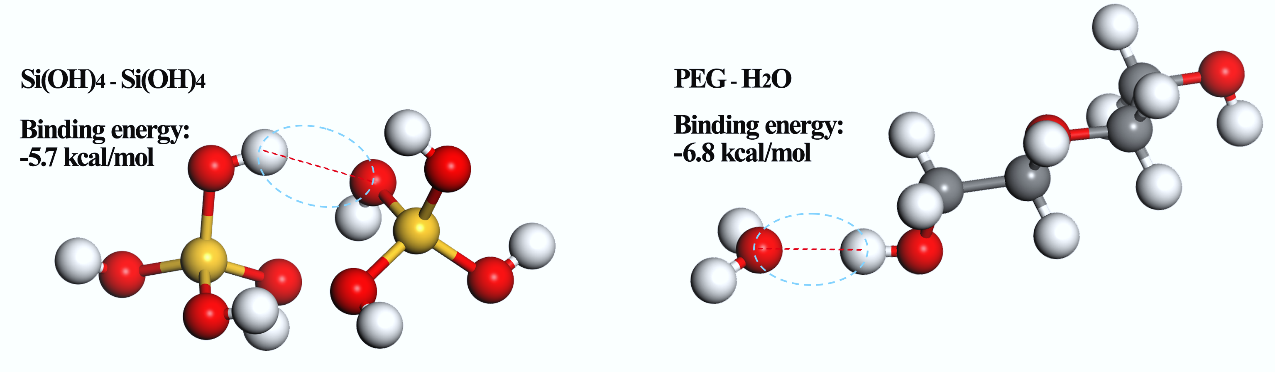


**Figure S2.** The electrostatic interactions between Si(OH)_4_, PEG and H_2_O units. In the TP sol system, the binding energies between PEG and either Si(OH)₄ or H₂O molecules are higher than the hydrogen bonding energy between –OH groups within Si(OH)₄ itself, which is only –5.7 kcal/mol. This gradient hydrogen bonding effect effectively delays the gelation time of the TPPCS, facilitating the formation of a uniform and stable phase change precursor.

**
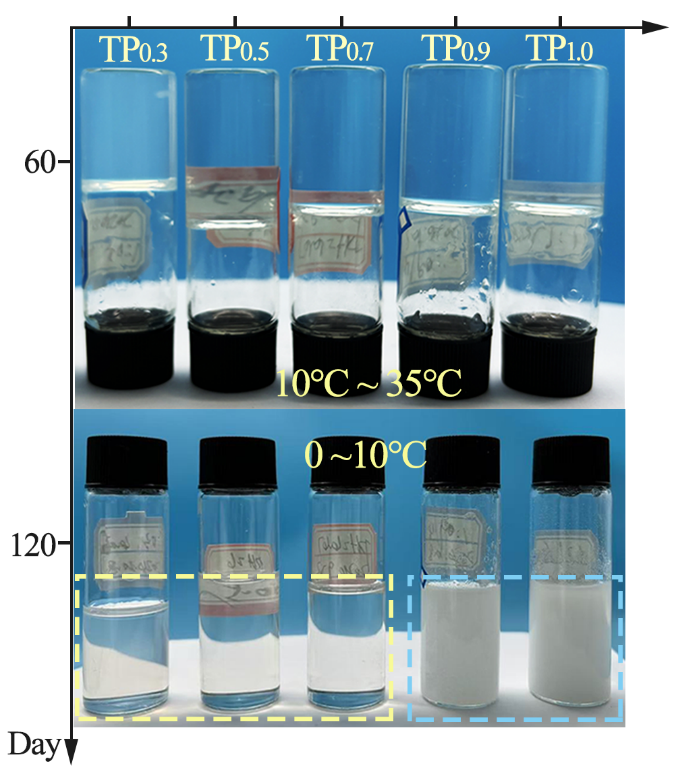
**

**Figure S3.** TP crystallization behavior at room temperature. By initiating the sol–gel process at elevated temperatures, a condensation reaction occurs, forming an organic–inorganic hybrid network in which PEG is embedded as a continuous phase within the three-dimensional structure, effectively achieving shape stabilization. Observations show that after gelation, the samples remained transparent when stored at room temperature (10–35 °C) for 60 days, indicating that the TP phase change composites can maintain a long-term supercooled state and effectively store latent heat at ambient conditions. However, as winter approached and the room temperature dropped to 0–10 °C, samples with TP_X≥0.9_ became opaque and underwent spontaneous crystallization, whereas samples with TP_X ≤ 0.7_ remained transparent, maintaining their supercooled state for up to 120 days or more. These results demonstrate that the temperature responsiveness of the composite phase change materials can be finely tuned by adjusting the composition ratio of the reaction system, enabling customized thermal regulation for different environmental conditions.


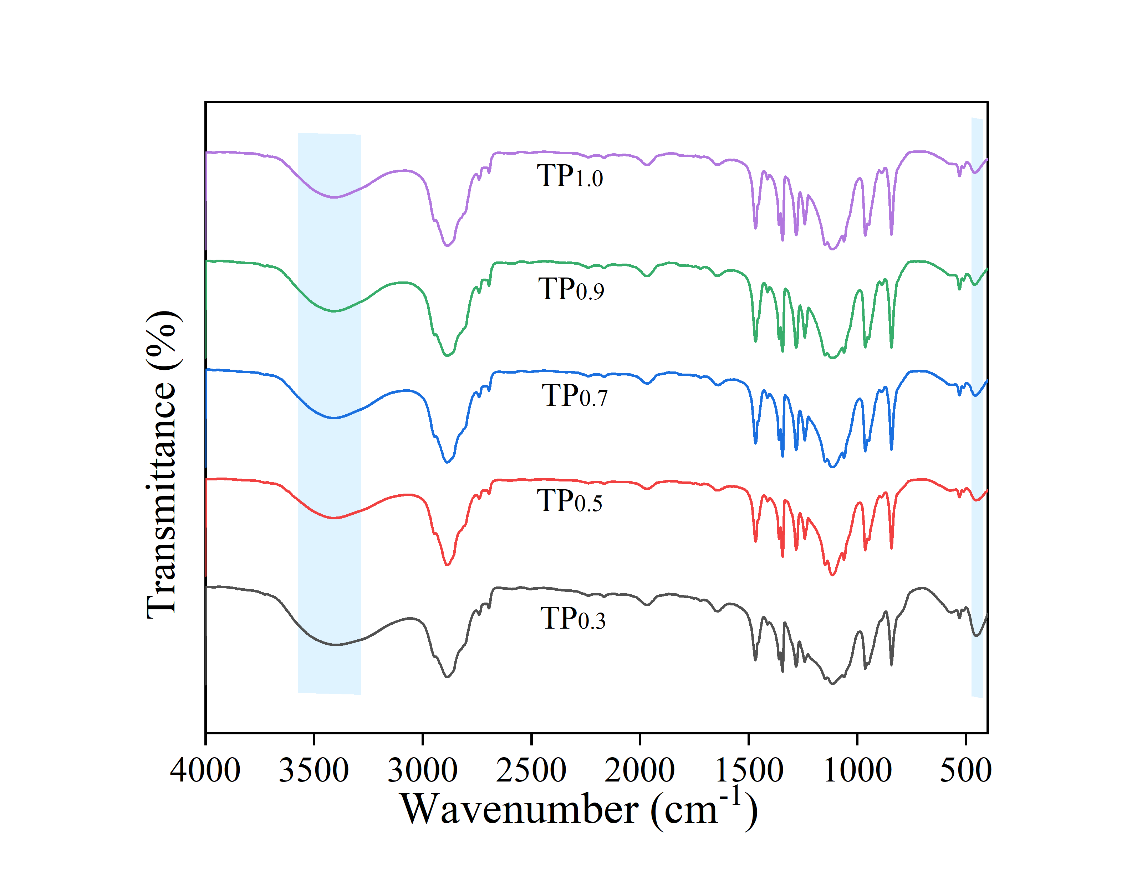


**Figure S4.** FT-IR of TP phase change composites with different mixing ratios. The bending vibrational peaks of Si-O-Si at 451 cm^-1^ of the samples are gradually enhanced with the increase of TEO ratio, indicating an increase in the density of the Si-O-Si network.


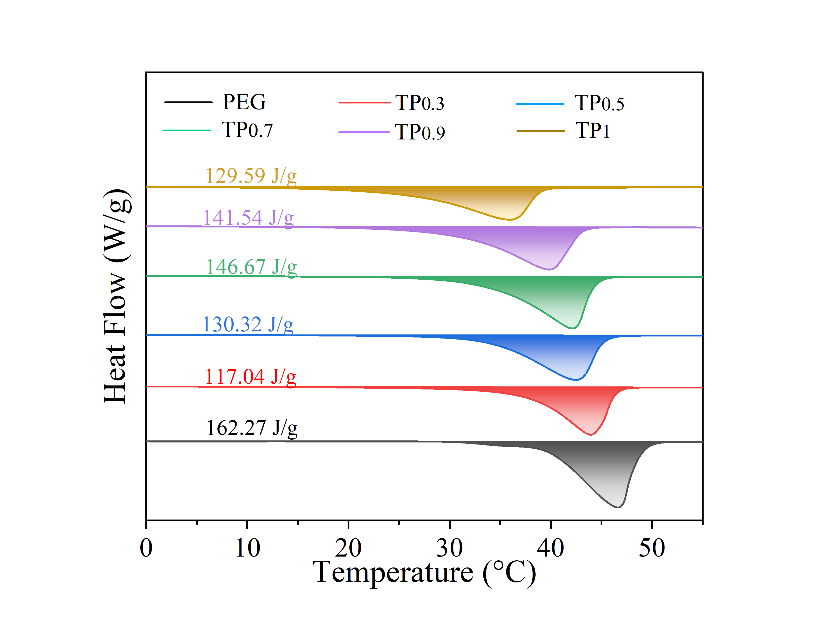

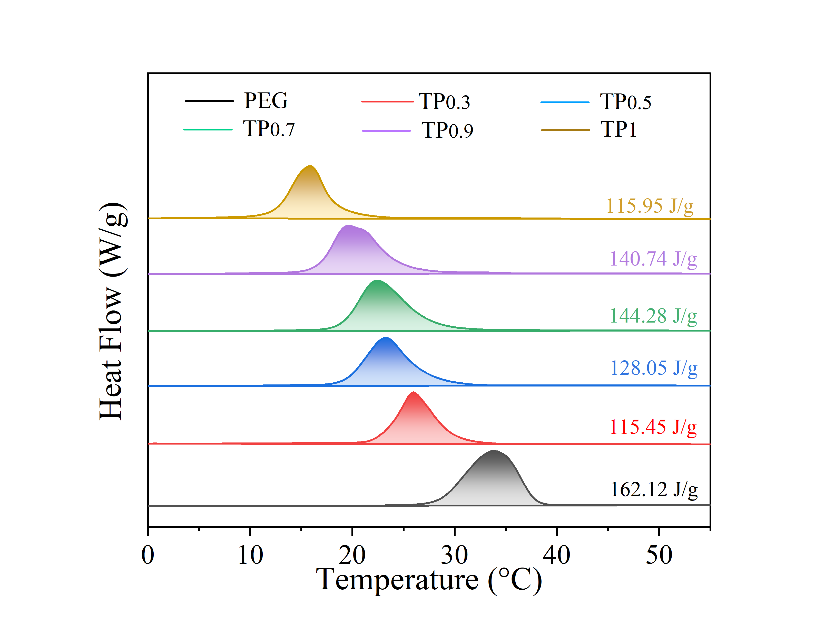


**Figure S5.** Crystallization (a) and melting (b) DSC curves of TP with different ratios.


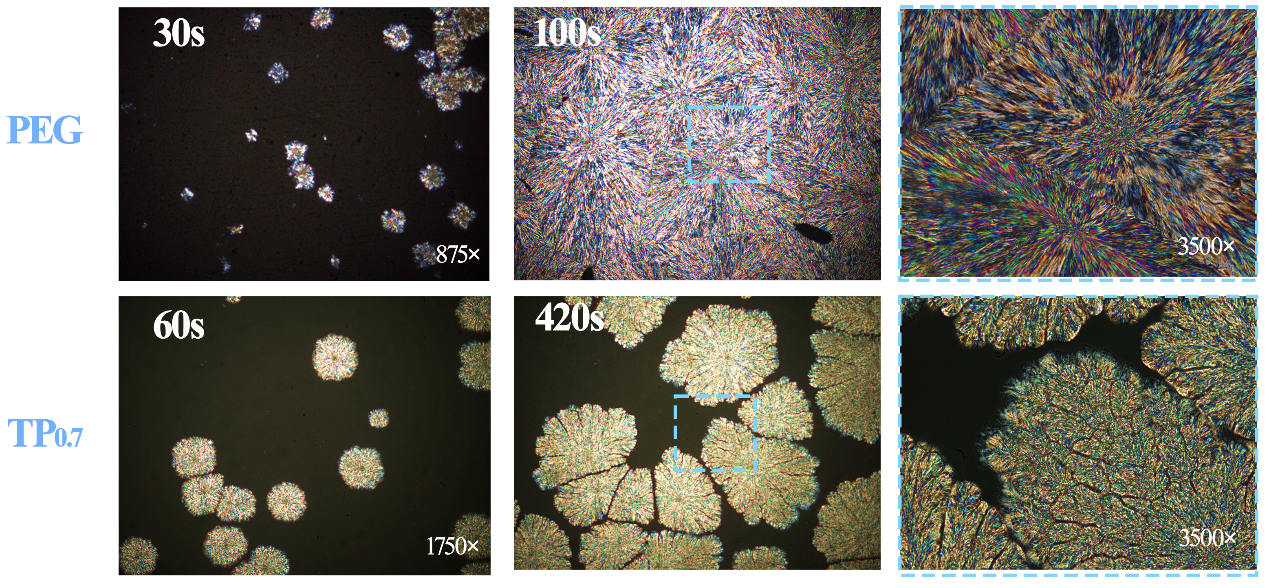


**Figure S6.** POM images of PEG, TP_0.7_ at 25 °C. Compared to pure PEG, both the spherulite size and crystallization rate of TP_0.7_ are suppressed. Moreover, microcracks are observed within the spherulites of TP_0.7_, which are likely caused by the uniformly embedded amorphous Si–O–Si network and hydrogen-bonding network within the PEG crystalline domains.


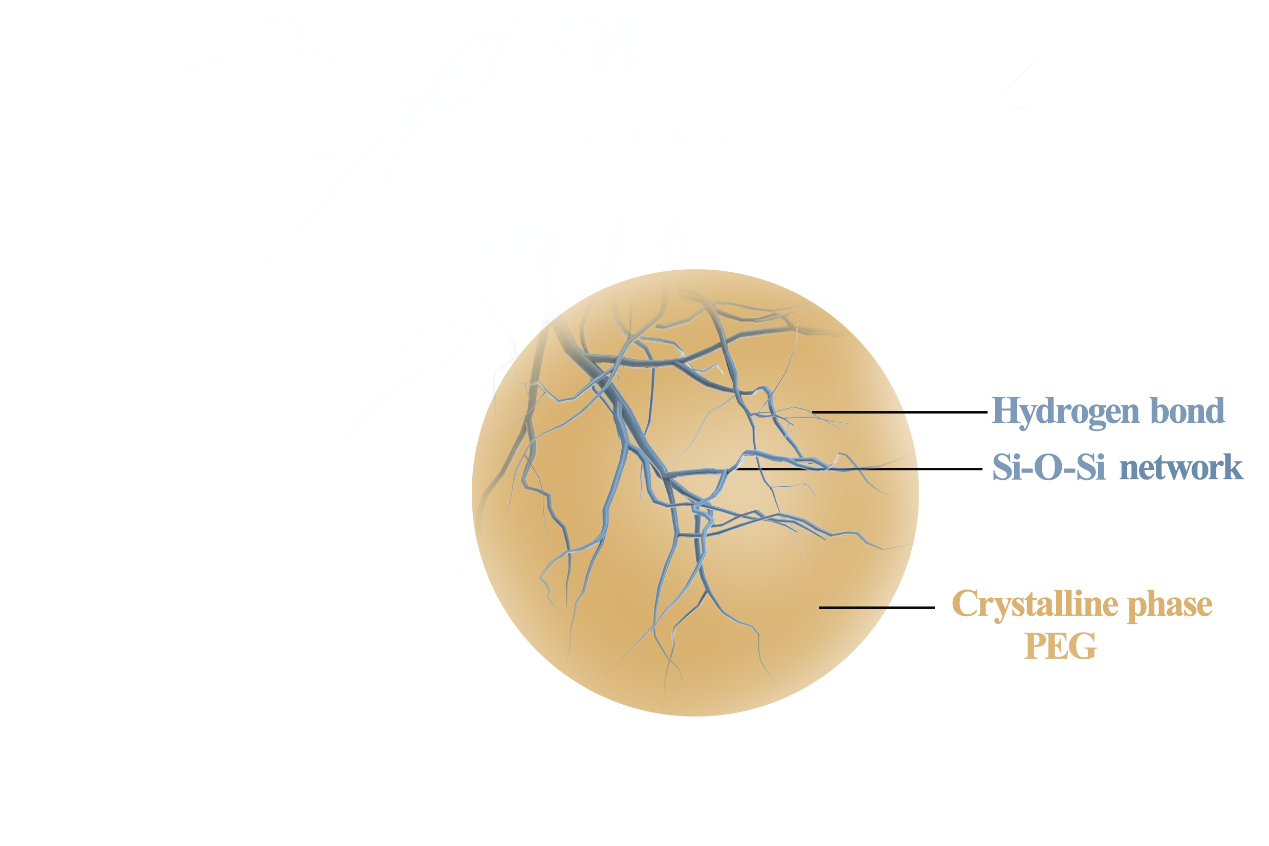


**Figure S7.** Schematic structure of crack formation by PEG crystallization.


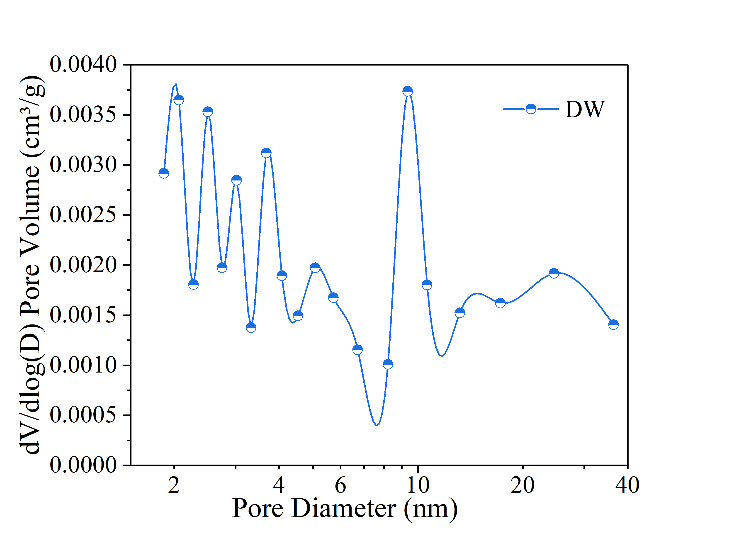

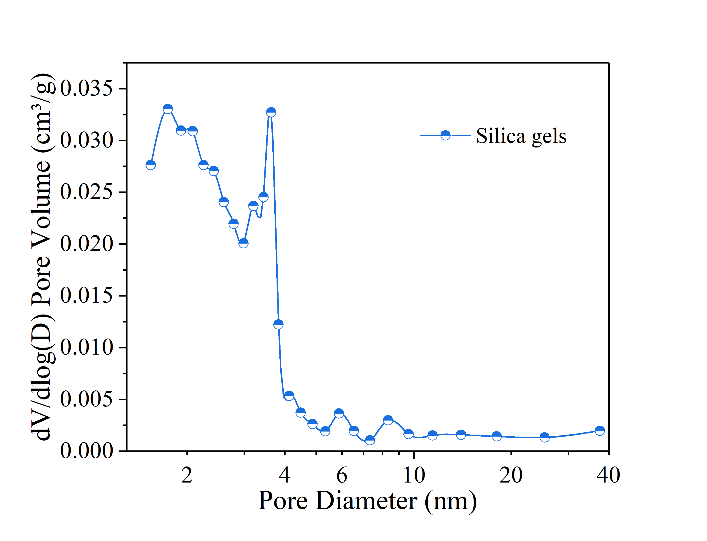


**Figure S8.** The pore size distributions of DW and Silica gels. Pore size distribution of DW derived from nitrogen adsorption–desorption isotherms using the BET method. The material exhibits a dominant mesoporous structure with a broad pore size distribution ranging from 2 to 40 nm. The larger and more interconnected pores in DW facilitate deeper infiltration and uniform dispersion of TPPCS, enabling the formation of an interlocked dual-network within the cellulose scaffold.


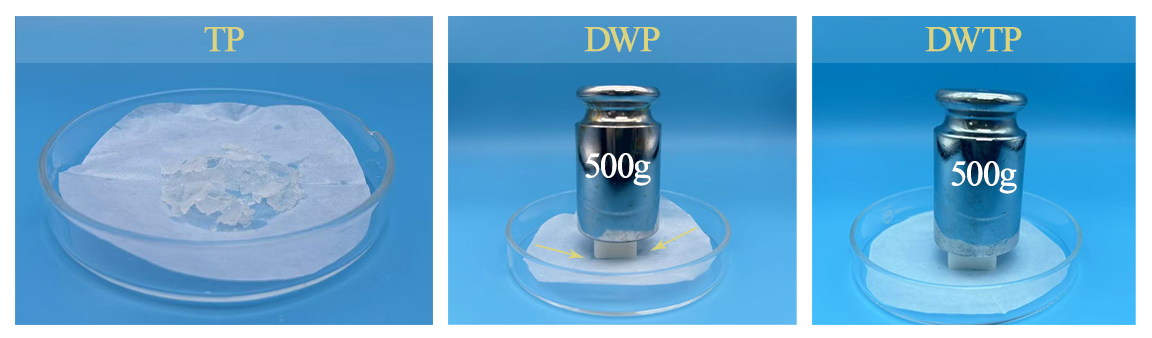


**Figure S9.** Leakage of TP, DWP, DWTP loaded with 500g and heated at 80℃ for 30 min. When the temperature reaches the melting point, TP undergoes significant deformation due to the absence of a structural support framework. In contrast, DWP exhibits no apparent macroscopic deformation thanks to the support provided by the wood scaffold, although partial PEG extrusion is observed. Benefiting from the synergistic effect of the cellulose skeleton, Si–O–Si covalent network, and hydrogen-bonding interactions, DWTP maintains its structural integrity without any leakage under a 500 g load at 80 °C for 30 minutes, highlighting the effectiveness of the interlocked dual-network architecture.

**
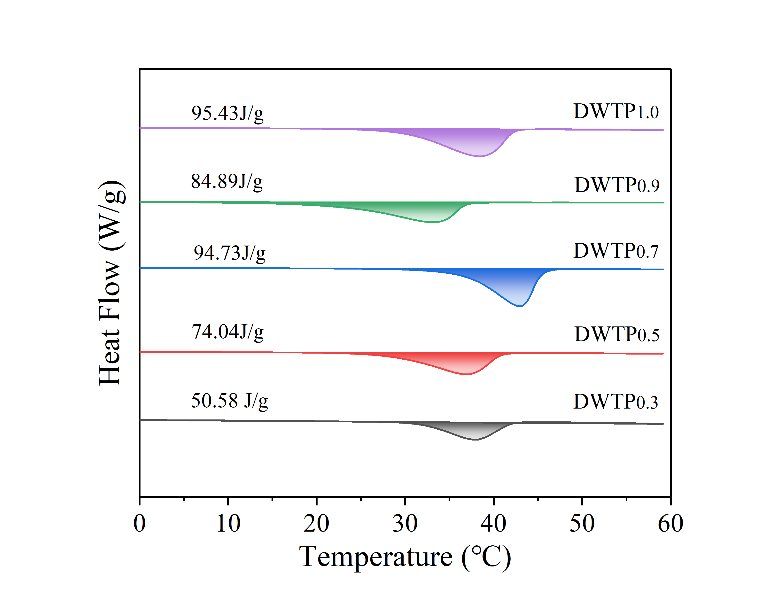

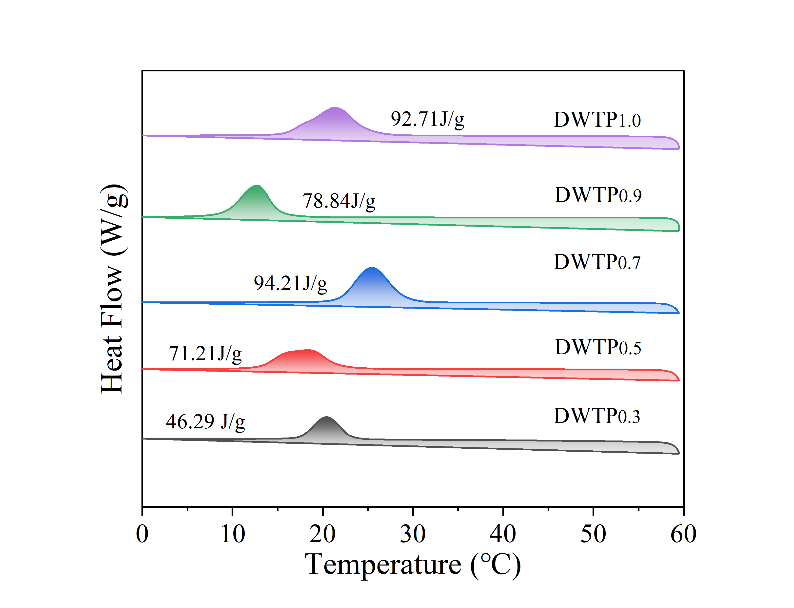
**

(b)

(a)

**Figure S10.** Crystallization (a) and melting (b) DSC curves of DWTP with different ratios.


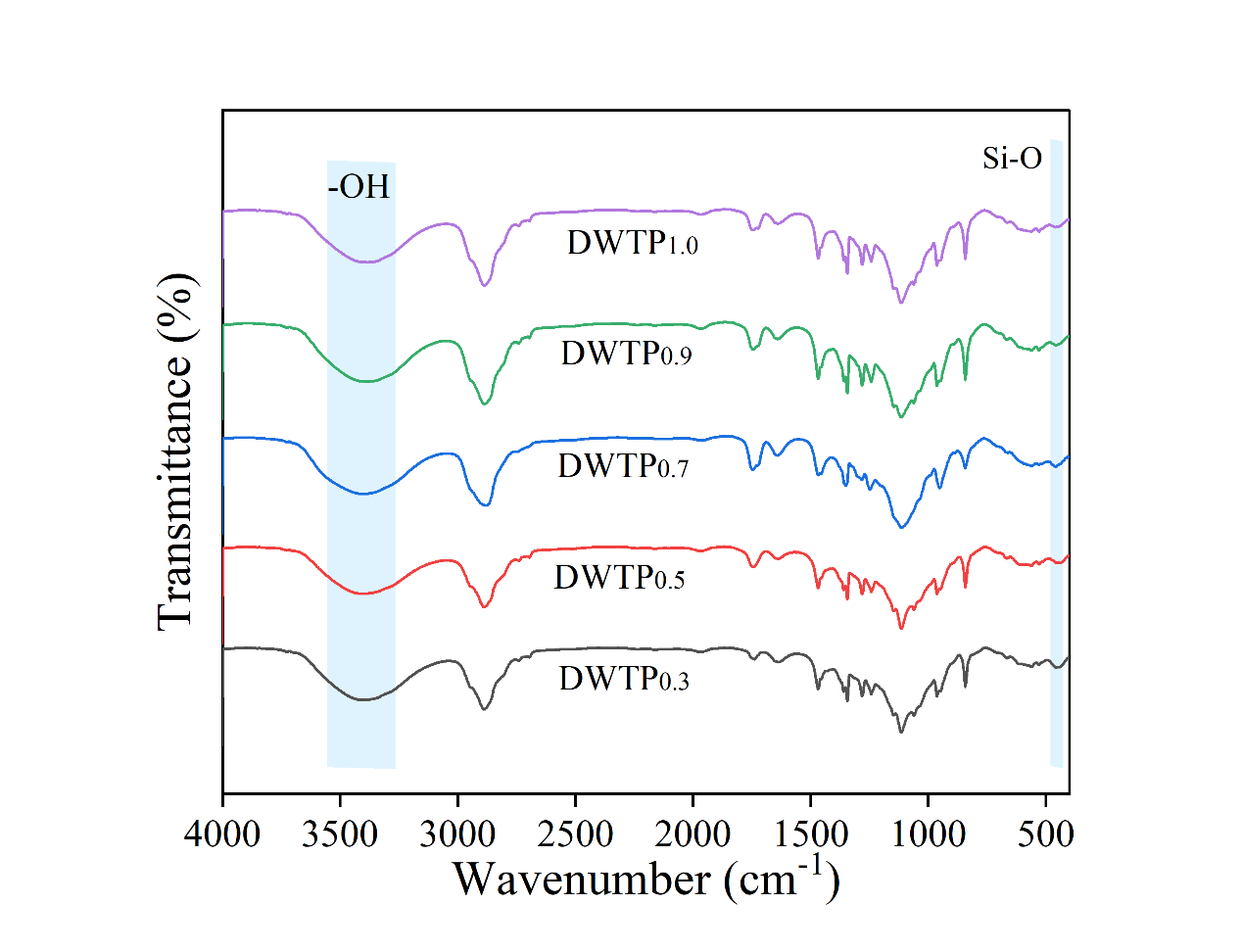


**Figure S11.** FT-IR of DWTP phase change composites with different ratios .


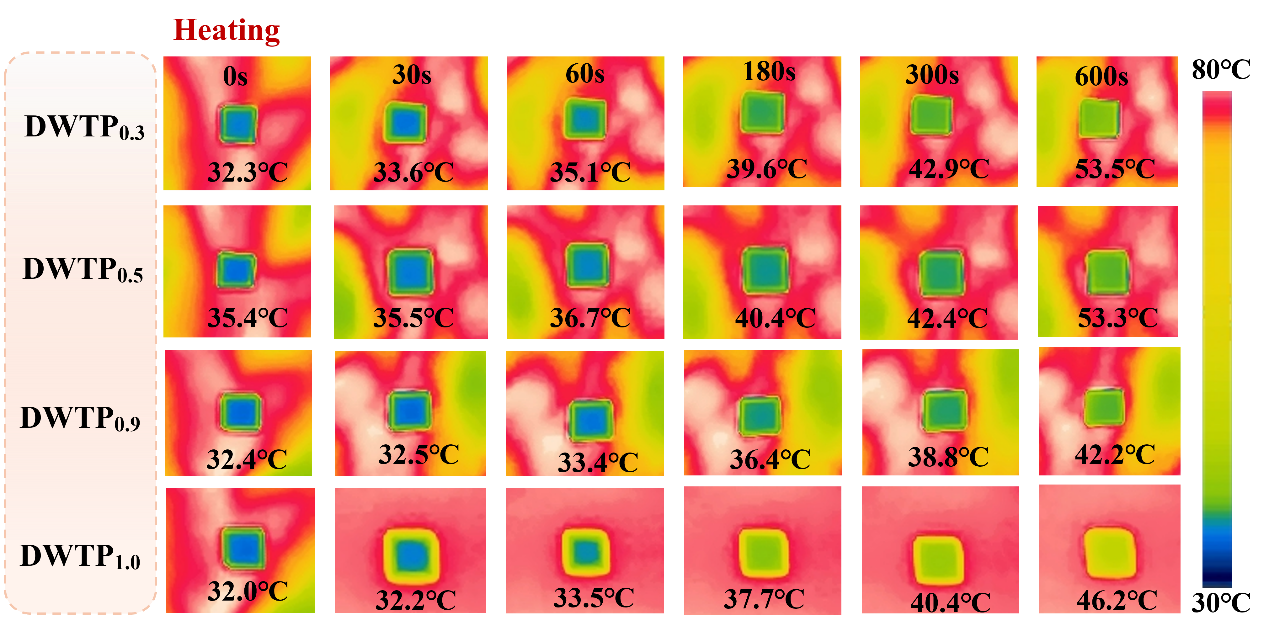


**Figure S12.** Thermal infrared imaging of DWTP samples during the heating. Over the same time period, the surface temperature of the samples decreased with increasing PEG content, which is attributed to the greater heat absorption resulting from the melting of larger amounts of PEG.

**
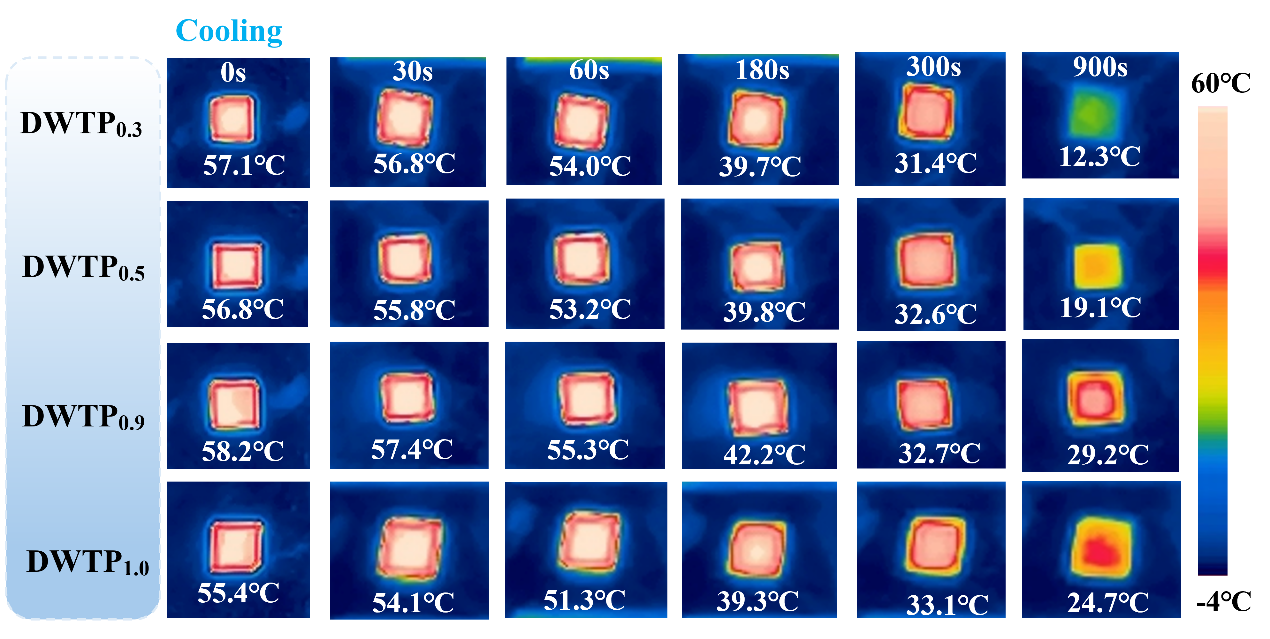
**

**Figure S13.** Thermal infrared imaging of DWTP samples during the cooling. At the same time, samples with higher PEG content exhibited slower cooling rates, indicating that DWTP can regulate temperature under cold shock conditions by releasing latent heat through PEG crystallization.


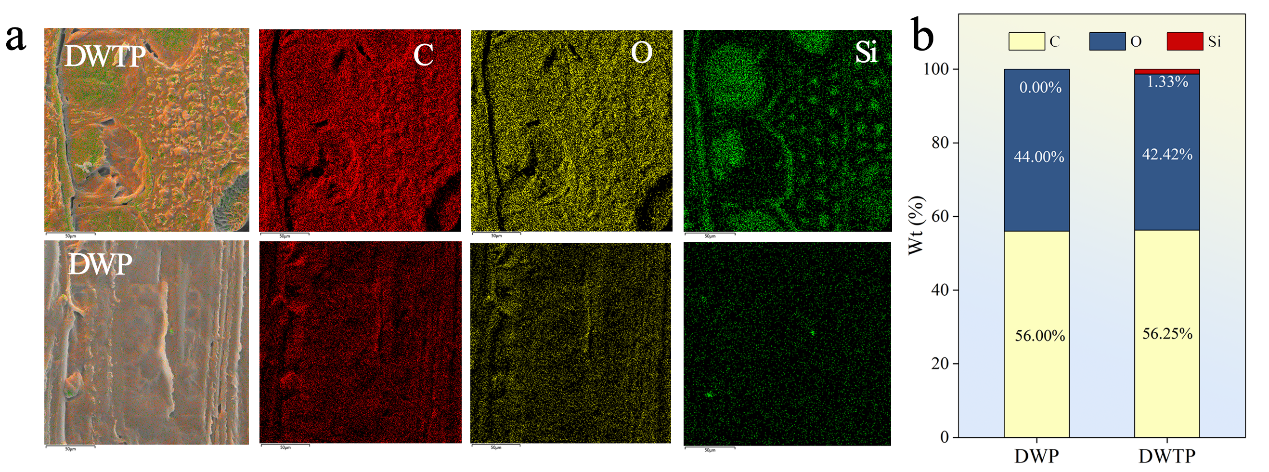


**Figure S14. (a)** EDS plots of DWP and DWTP. (b) Elemental mass ratios of DWP and DWTP. Compared to DWP, DWTP exhibits a faster thermal response rate, which may be attributed to the Si-O-Si network embedded within the PEG.


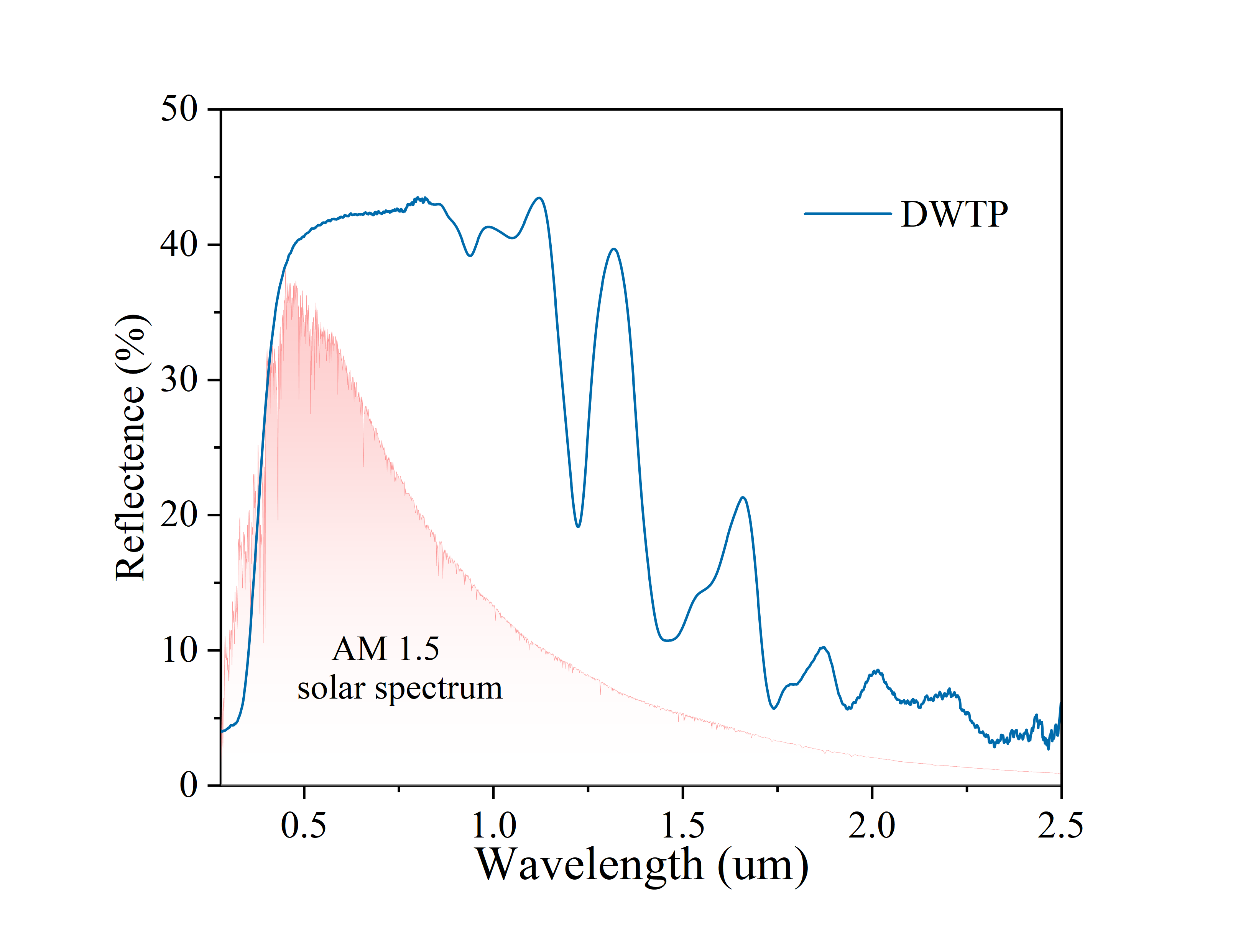


**Figure S15.** Solar reflectance of DWTP. The solar radiation intensity weighted reflectance of DWTP is 40.3% (relative to the AM1.5 solar spectrum), effectively reducing the surface temperature of the sample under solar irradiation. This effect primarily originates from the intrinsic chemical bonding characteristics of DWTP. Specifically, the C–O–C and C–O bonds in the cellulose framework of wood, and the Si–O–Si bonds formed through the condensation of TPPCS, exhibit high selective emissivity, thereby effectively enhancing the radiative cooling performance^[1-2]^.

**Ⅲ. Supplementary Table**

**Table S1. Different reaction parameters**

| **Sample** | **TEOs (mol)** | **PEG (mol)** | **pH** | **leakage rate (%)** |
| --- | --- | --- | --- | --- |
| PEG | 0 | 1 | / | 100.00 |
| DWP | 0 | 1 | / | 1.22 |
| TP_0.7_ | 1 | 0.7 | 4.03 | 1.31 |
| TP_0.7_ | 1 | 0.7 | 4.47 | 11.33 |
| TP_0.7_ | 1 | 0.7 | 5.14 | 11.66 |
| TP_0.3_ | 1 | 0.3 | 4.03 | 7.00 |
| TP_0.5_ | 1 | 0.5 | 4.03 | 7.77 |
| TP_0.7_ | 1 | 0.7 | 4.03 | 1.18 |
| TP_0.9_ | 1 | 0.9 | 4.03 | 4.25 |
| TP_1.0_ | 1 | 1 | 4.03 | 8.60 |
| DWTP_0.7_ | 1 | 0.7 | 4.03 | 0.04 |

**Table S2. Comparison of phase change and mechanical properties with those of the corresponding references.**

| **Supporting Materials** | | **PCM** | **T_m_/T_c_ (℃)** | **ΔH (J/g)** | **Tensile stress (Mpa)** | **Refs** |
| --- | --- | --- | --- | --- | --- | --- |
| Polymer | PUA | Myristic acid | 52.7-55.3 | 113.4 | 25-34 | Ref.1 |
|  | TPEE | Paraffin | 32.2-48.4 | 112.3-145.9 | 4.1-7.3 | Ref.2 |
|  | PUA | PEG | 28;42 | 113.1 | 5 | Ref.3 |
|  | AMD-HDI | PEG | 38.8-51.1 | 79.7-116.7 | 15 | Ref.4 |
|  | BQDO-IPDI | PEG | 15-38 | 54.4-86.1 | 12-30 | Ref.5 |
|  | HMAU-HDI | PEG | 46.4;57.9 | 142.5 | 36.9 | Ref.6 |
|  | EP-CNT | Beeswax | 57.7 | 72.2 | 8.6 | Ref.7 |
| Aerogel,  foam | Diatomite | PEG | 27.7;32.2 | 87.1 | / | Ref.8 |
|  | CNT/MXene | Eucommia ulmoides gum | 12.5;48.6 | 36.8 | / | Ref.9 |
|  | PLA | Paraffin | 60.62;70.31 | 199.3 |  | Ref.10 |
|  | PVA-CNC | PEG | 32 | 44.2 | / | Ref.11 |
|  | PVA-PLA-MXene | PCC | 35;45 | 104.5-141.4 | 0.5-4 | Ref.12 |
|  | Cellulose | PEG | -5.4;7.7 | 78.6 | / | Ref.13 |
|  | CMA-HEMA | PEG | 22.3;41.1 | 90.6 | 0.4 | Ref.4 |
| Wood | Delignified wood | Capric acid-Palmitic acid | 14.5;23.4 | 94.4 | / | Ref.15 |
|  | Man- Delignified wood | PEG-GMA | 10.7;25.5 | 25.1 | / | Ref.16 |
|  | Wood-PVA | PEG | 33.1;55.8 | 135.4 | 80.9 | Ref.17 |
|  | Carbonized delignified  wood | PEG | 60.7;27.9 | 151.74 | / | Ref.18 |
|  | Delignified wood | Dodecanol | 16.5;25.7 | 83.2 | 60.8 | Ref.19 |
|  | Wood | Myristic acid | 51.0;53.5 | 26.1 | / | Ref.20 |
|  | Delignified wood | PEG | 25.4;42.3 | 94.7 | 134.4 | This work |

**Table S3. Comparisons of the thermal properties of PEG and composite phase change material.**

| **Sample** | **ΔH_m_ (J/g)** | **T_m_(℃)** | **ΔH_c_ (J/g)** | **T_c_(℃)** | **Refs** |
| --- | --- | --- | --- | --- | --- |
| PEG1500 | 168.2 | 43.6 | 158.4 | 26.9 | Ref.21 |
| W-P | 95.0 | 39.8 | 83.7 | 28.6 |  |
| PEG4K | 183.8 | 53.2 | 178.7 | 36.8 | Ref.6 |
| PEG10K | 180.9 | 60.9 | 177.3 | 38.5 |  |
| PEG20K | 176.3 | 61.9 | 172.1 | 41.4 |  |
| MHPCM-4K | 90.0 | 39.7 | 87.6 | 24.7 |  |
| MHPCM-10K | 97.2 | 45.1 | 96.0 | 34.5 |  |
| MHPCM-20K | 142.5 | 57.9 | 140.1 | 46.4 |  |
| PW | 227.8 | 45.4 | 228.3 | 41.6 | Ref.22 |
| OBC-SEBS-PW | 175±5 | 41±1 | 175±5 | 41±1 |  |
| SAT | 254.2 | 58.4 | / | / | Ref.23 |
| PCH | 179.2 | 57.21 | / | / |  |
| PEG400 | 171.2 | 7.2 | 157.8 | -16.4 | Ref.17 |
| PEG1K | 167.7 | 40.5 | 168.3 | 27.5 |  |
| PEG2K | 182.9 | 55.9 | 166.6 | 34.2 |  |
| PEG4K | 213.1 | 63.5 | 177.7 | 43.0 |  |
| PEG400/PVA/W | 42.2 | 2.29 | 17.8 | -17.3 |  |
| PEG1K/PVA/W | 68.1 | 39.9 | 69.7 | 29.1 |  |
| PEG2K/PVA/W | 135.4 | 55.8 | 118.4 | 33.1 |  |
| PEG4K/PVA/W | 121.3 | 60.6 | 98.2 | 41.4 |  |
| DWTP | 94.7 | 42.9 | 94.2 | 25.4 | This work |

**Supplementary References**

1. Li C, et al. A novel one-step ultraviolet curing fabrication of myristic acid-resin shape-stabilized composite phase change material for low temperature thermal energy storage. Chem. Eng. J. 458, 141355 (2023).

2. Zhao X, et al. A shape-memory, room-temperature flexible phase change material based on PA/TPEE/EG for battery thermal management. Chem. Eng. J. 463, 142514 (2023).

3. Zhang Q, et al. Polyethylene glycol/polyurethane acrylate-based flexible phase- change film with excellent mechanical strength and reversible optical performance. Energy Fuels 37, 3227-3235 (2023).

4. Deng C, et al. Synchronous visual/infrared stealth using an intrinsically flexible self‐healing phase change film. Adv. Funct. Mater. 33, 2212259 (2023).

5. Yang Y, et al. A novel intrinsic photothermal and flexible solid-solid phase change materials with super mechanical toughness and multi-recyclability. Appl. Energy 332, 120564 (2023).

6. Wang C, et al. Multiple h-bonding cross-linked supramolecular solid–solid phase change materials for thermal energy storage and management. Adv. Mater. 36, 2309723 (2024).

7. Fan Y, et al. Relationship between cross-linking network structure and phase change performances toward multifunctional epoxy/bio-based wax form-stable phase change materials. Chem. Eng. J. 454, 140221 (2023).

8. Karaman S, et al. Polyethylene glycol (PEG)/diatomite composite as a novel form-stable phase change material for thermal energy storage. Sol. Energy Mater. Sol. Cells. 95, 1647 (2011).

9. Han L, et al. Bio-based, phase-change MXene/CNT foams for integrated electromagnetic interference shielding, thermal management and infrared stealth. Adv. Nanocomposites. 2, 148-161 (2025).

10. Yin G, et al. PLA aerogel as a universal support for the typical organic phase change energy storage materials. J. Energy Storage. 73, 108869 (2023).

11. Wang F, et al. Bioinspired biodegradable sandwich‑structured porous metafabric for passive personal thermal management. Adv. Fiber Mater. (2025).

12. Li X, et al. Wearable janus-type film with integrated all-season active/passive thermal management, thermal camouflage, and ultra-high electromagnetic shielding efficiency tunable by origami process. Adv. Funct. Mater. 33, 2212776 (2023).

13. Özgül Gök, et al. Developing a poly(ethylene glycol)/cellulose phase change reactive composite for cooling application. Sol. Energy Mater. Sol. Cells. 191, 345-349 (2019).

14. Meng D, et al. Carboxymethyl cellulose enhanced polymeric form stable phase change materials with reversible transparency for energy storage. ACS Sustainable Chem. Eng. 42, 15623–15633 (2024).

15. Ma L, et al. Delignified wood/capric acid-palmitic acid mixture stable-form phase change material for thermal storage. Sol. Energy Mater. Sol. Cells. 194, 215-221 (2019).

16. Li Y, et al. Processing wood into a phase change material with high solar-thermal conversion efficiency by introducing stable polyethylene glycol-based energy storage polymer. Energy. 254, 124206 (2022).

17. Liu Y, et al. Muscle-inspired formable wood-based phase change materials. Adv. Mater. 2406915 (2024).

18. Li Y, et al. Enzymolysis-treated wood-derived hierarchical porous carbon for fluorescence-functionalized phase change materials. Compos. Part B-Eng. 234, 109735 (2022).

19. Zhou J, et al. Phase-change thermal storage transparent wood based on optical reversibility. Adv. Funct. Mater. 223, 120275 (2025).

20. Ahmet C, et al. Properties of Pinus nigra Arn. wood impregnated with phase change materials for potential energy-saving building material. J. Energy Storage. 83, 110687 (2024).

21. Meng Y, et al. Form-stable phase change materials from mesoporous balsa after selective removal of lignin. Compos. Part B-Eng. 199, 108296 (2020).

22. Jing Y, et al. Ultraflexible, cost-effective and scalable polymer-based phase change composites via chemical cross-linking for wearable thermal management. Nat. Commun. 14, 8060 (2023).

23. Song M, et al. Thermally induced flexible phase change hydrogels for solar thermal storage and human thermal management. Chem. Eng. J. 464, 142682 (2023).
